# ADAPTations to low load blood flow restriction exercise versus conventional heavier load resistance exercise in UK military personnel with persistent knee pain: protocol for the ADAPT study, a multi-centre randomized controlled trial

**DOI:** 10.1186/s12891-023-06693-3

**Published:** 2023-07-17

**Authors:** Robyn P. Cassidy, Kieran M. Lunt, Russell J. Coppack, Alexander N. Bennett, James L. J. Bilzon, M. Polly Mcguigan, Natalie Egginton, Edward Sellon, Jo Day, Peter Ladlow

**Affiliations:** 1 Academic Department of Military Rehabilitation (ADMR), Defence Medical Rehabilitation Centre (DMRC) Stanford Hall, Loughborough, LE12 5BL UK; 2 Versus Arthritis Centre for Sport, Exercise and Osteoarthritis Research, Department for Health, University of Bath, Bath, UK; 3 Department for Health, University of Bath, Bath, UK; 4 National Heart and Lung Institute, Imperial College London, London, UK; 5 Royal Centre for Defence Medicine (RCDM), Birmingham, UK; 6 Radiology Department, Defence Medical Rehabilitation Centre (DMRC) Stanford Hall, Loughborough, UK

**Keywords:** Occlusion training, Defence rehabilitation, Strength training, Training load, Musculoskeletal health, Knee injury, Lower limb, Occupational rehabilitation, Return-to-duty

## Abstract

**Background:**

Muscle atrophy, muscle weakness and localised pain are commonly reported following musculoskeletal injury (MSKI). To mitigate this risk and prepare individuals to return to sport or physically demanding occupations, resistance training (RT) is considered a vital component of rehabilitation. However, to elicit adaptations in muscle strength, exercise guidelines recommend lifting loads ≥ 70% of an individual’s one repetition maximum (1-RM). Unfortunately, individuals with persistent knee pain are often unable to tolerate such high loads and this may negatively impact the duration and extent of their recovery. Low load blood flow restriction (LL-BFR) is an alternative RT technique that has demonstrated improvements in muscle strength, hypertrophy, and pain in the absence of high mechanical loading. However, the effectiveness of high-frequency LL-BFR in a residential rehabilitation environment remains unclear. This study will compare the efficacy of high frequency LL-BFR to ‘conventional’ heavier load resistance training (HL-RT) on measures of physical function and pain in adults with persistent knee pain.

**Methods:**

This is a multicentre randomised controlled trial (RCT) of 150 UK service personnel (aged 18–55) admitted for a 3-week residential rehabilitation course with persistent knee pain. Participants will be randomised to receive: a) LL-BFR delivered twice daily at 20% 1-RM or b) HL-RT three-times per week at 70% 1-RM. Outcomes will be recorded at baseline (T1), course discharge (T2) and at three-months following course (T3). The primary outcome will be the lower extremity functional scale (LEFS) at T2. Secondary outcomes will include patient reported perceptions of pain, physical and occupational function and objective measures of muscle strength and neuromuscular performance. Additional biomechanical and physiological mechanisms underpinning both RT interventions will also be investigated as part of a nested mechanistic study.

**Discussion:**

LL-BFR is a rehabilitation modality that has the potential to induce positive clinical adaptations in the absence of high mechanical loads and therefore could be considered a treatment option for patients suffering significant functional deficits who are unable to tolerate heavy load RT. Consequently, results from this study will have a direct clinical application to healthcare service providers and patients involved in the rehabilitation of physically active adults suffering MSKI.

**Trial registration:**

ClinicalTrials.org reference number, NCT05719922

**Supplementary Information:**

The online version contains supplementary material available at 10.1186/s12891-023-06693-3.

## Background

The goal of most rehabilitation care pathways is to safely return a patient to their previous level of physical function. It is widely acknowledged that muscle atrophy can prolong the duration of musculoskeletal rehabilitation, increase the cost to healthcare providers and prevent optimal recovery [1]. A significant challenge lies in designing optimal rehabilitation programmes that facilitate both neurological and muscular adaptations whilst accommodating for biological healing and patient safety [2]. Symptomatic impairment, including immobility and pain, may limit an individual’s ability to tolerate the heavy-load resistance training (RT) methods often recommended to elicit increases in muscle strength and hypertrophy [3, 4]. Therefore, patients with musculoskeletal injury (MSKI), such as persistent knee pain, are often advised to reduce their training load, potentially limiting the desired muscular response to treatment and delaying their subsequent return to sport or occupational roles.

Knee pain can arise from the tibiofemoral joint and/or the patellofemoral joint, with common diagnosed pathologies including osteoarthritis (OA), meniscal pathology and patellofemoral pain [5–7]. Persistent knee pain has been associated with decreased function and physical activity levels and consequently can lead to progressive muscle atrophy [8, 9]. Subsequently, a negative feedback response can occur as decreased muscle strength limits activity participation leading to further muscular deterioration and exacerbation of symptoms [10, 11]. Thus, addressing injury related muscle atrophy is a vital part of rehabilitation for persistent knee pain [12–14].

Within military cohorts, MSKI is the leading cause of medical downgrading and discharge [15–17]. This can negatively impact operational readiness, place an increased demand on global Defence healthcare system and is associated with considerable economic cost [18, 19]. Injury and pain at the knee joint constitute a significant proportion of all reported MSKI [20–23]. In Europe, one-fifth of serving personnel report knee pain during their military career. Additionally, in the American military, knee injury is the most frequent reason for hospitalisation [20, 22]. Within the UK armed forces, knee pain accounted for 17–25% of all MSKI related medical discharges in 2021/2022 [17].

Within UK Defence, serving personnel with persistent knee pain are offered a 3-week intensive residential rehabilitation course at a regional rehabilitation unit (RRU). These multidisciplinary courses have demonstrated efficacy for improving clinical outcomes in a variety of MSKI [24–26]. For military personnel, regaining muscular capacity is deemed a priority within rehabilitation to improve a soldiers’ tolerance and performance in common physically demanding military-based tasks [27]. Traditional guidelines recommend that RT programmes incorporate strengthening exercises with loads of ≥ 70% 1-repetition maximum (1-RM) [4]. However, for many service personnel undergoing rehabilitation, heavy loads cannot be tolerated due to pain and functional limitations [28].

An alternative RT approach is low load blood flow restriction (LL-BFR) training. With this method, a pneumatic tourniquet system is applied to the proximal region of a limb and inflated to determine their personalised limb occlusion pressure (LOP). A percentage of this LOP is then prescribed (often 80% LOP) which will partially reduce arterial inflow, but fully occlude venous outflow; exercises are then performed under low load, typically 20–40% 1-RM [29]. The compression of vasculature, coupled with skeletal muscle contraction during LL-BFR is known to increase the metabolic stress associated with exercise [30, 31]. This increase in metabolic stress is believed to induce hypertrophic adaptations through several mechanisms, such as an increase in the recruitment of fast-twitch muscle fibres [32] and by increasing intra-cellular water content (cell swelling), which could increase protein synthesis and decrease protein breakdown [33, 34]. Indeed, significant improvements in both muscle strength and hypertrophy, similar to that of traditional heavier load RT approaches have been demonstrated using LL-BFR in healthy individuals [35–38]. To date however studies have restricted their measures of muscle hypertrophy to measures of anatomical cross-sectional area (ACSA) and/or volume, with no research in clinical populations investigating the effect of LL-BFR on the physiological cross-sectional area (PCSA), the only architectural measurement which is known to directly influence tetanic force production [39].

Improvements in muscular capacity in the absence of, or reduced, mechanical loading have led to increasing interest in the use of LL-BFR in the rehabilitation setting [40–44]. Positive clinical adaptations for muscle strength, hypertrophy and self-reported function following a course of LL-BFR have been shown in knee OA [45, 46], patellofemoral pain [47] and post ACL reconstruction [48]. In addition, reduced levels of pain have also been observed following a single bout [49] and repeated bouts of LL-BFR [47]. This exercise induced hypoalgesia response makes LL-BFR a particular clinical interest when rehabilitating symptomatic MSKI pain [50, 51].

The lower relative training loads and consequential lower mechanical stress during exercise may reduce associated exercise induced muscle damage (EIMD). Here, indirect markers of EIMD, such as torque decrements, delayed onset of muscle soreness (DOMS), range of movement (ROM), creatine kinase (CK) and myoglobin are typically lower following an acute bout of LL-BFR compared to conventional high load RT [29, 52, 53]. This may reduce the requirement for long inter session recovery periods and thus allow for an intensive course of treatment to be implemented. Favourable muscle adaptations following high frequency training protocols have been documented. Notably, increases in lower limb strength and muscle cross sectional area (CSA) following twice daily treatments over a period of 2–3 weeks [54–58]. A recent scoping review of short-term, high frequency BFR training protocols have identified numerous limitations within the existing literature. The ADAPT study will help to address many of the knowledge gaps identified, including; 1) the lack of personalised pressure applications, 2) lack of females, 3) lack of studies using clinical populations, 4) potentially high numbers of under-powered study designs, 5) limited data regarding the proximal effects of BFR, and 6) the absence of data investigating hypoalgesia responses to high frequency [59].

The effectiveness of a high frequency training approach is of particular interest to professional sport teams and providers of residential rehabilitation (e.g., UK Defence Rehabilitation). A pilot study randomised controlled trial (RCT) assessing the feasibility and acceptability of introducing twice daily LL-BFR within a busy UK Defence residential rehabilitation setting has previously been demonstrated [26]. LL-BFR was found to produce similar training adaptations in relation to muscle strength, hypertrophy and physical function when compared to conventional rehabilitation. However, it is unclear if the observed improvements in muscle strength translated into performance improvements during occupational specific tasks requiring high levels of force production, or alterations in kinetic or kinematic variables during functional movements. Furthermore, as Ladlow et al. [26] did not include any longer-term outcome measures following either treatment intervention, it is not possible to speculate on the potential longer-term benefits of LL-BFR beyond the 3-week rehabilitation intervention.

Pragmatic research trials are designed to assess the effectiveness of an intervention as it would be delivered in the ‘real world’, rather than under highly controlled conditions. More pragmatic research trials, embedded within the rehabilitation setting, are needed to advance our understanding of the benefits of LL-BFR based therapeutic interventions. This study consists of two distinct but inter-dependent experimental approaches; a multicentre RCT and a nested mechanistic based study.

### Study aims

The aim of the main RCT and the nested mechanistic study is to investigate the effects of high frequency, low-load resistance training using blood flow restriction (LL-BFR) and ‘conventional’ heavier load resistance training (HL-RT) in UK military personnel with persistent knee pain.

#### Main RCT

1. The main RCT will compare the effects of the two interventions on physical function and pain.

#### Nested mechanistic study

2) The nested mechanistic study will compare the effects of the two interventions on i) muscle morphology and architecture, ii) muscle strength, iii) performance during occupational specific tasks, iv) kinetic and kinematic variables during functional tasks and v) blood biomarkers of EIMD, inflammation, oxidative stress and vascular function.

## Methods

### Design

**Fig. 1:**
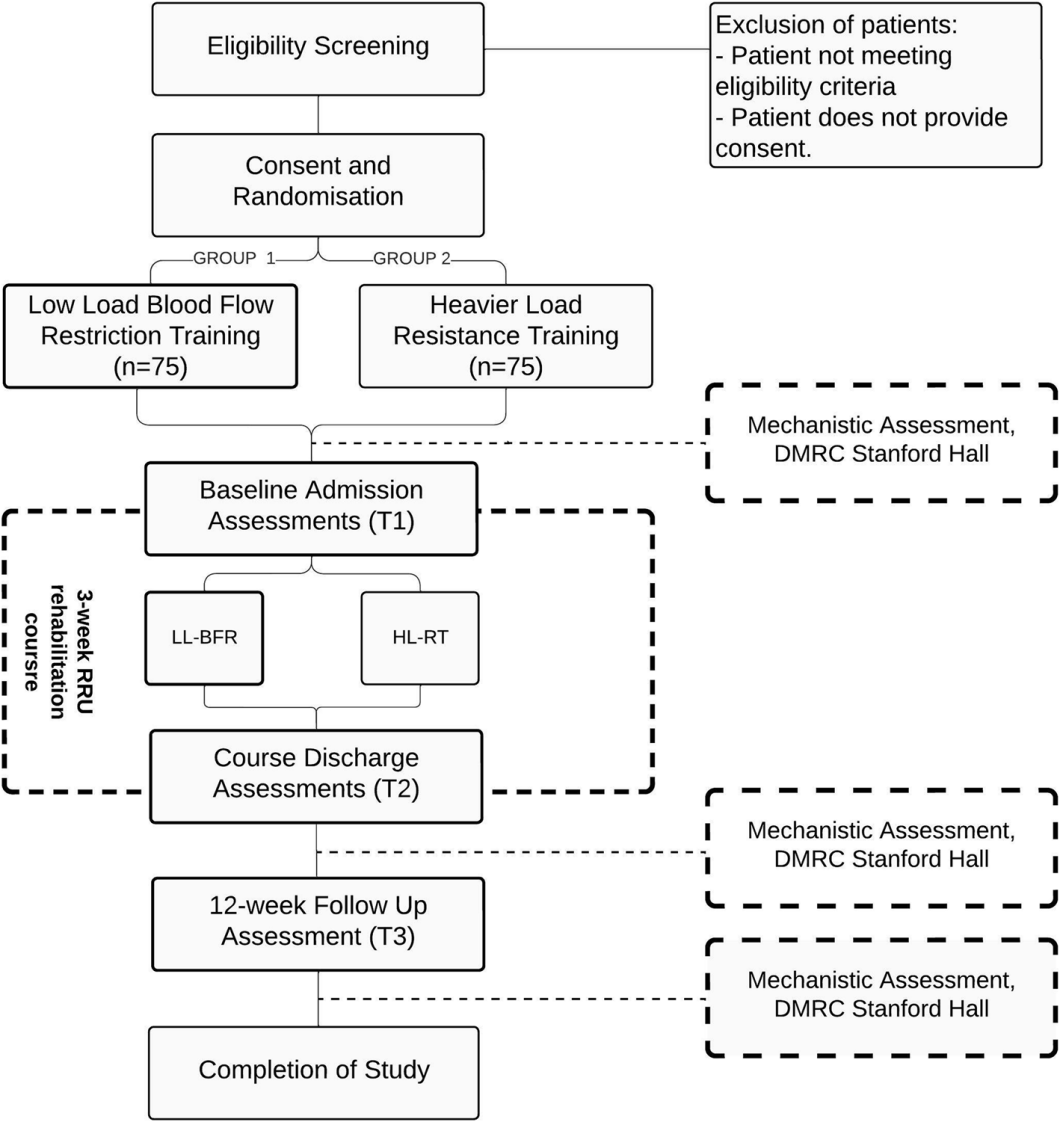
Study design flow diagram

This is a prospective, multicentre RCT embedded within five UK Defence RRUs. Serving personnel will be randomly assigned to one of the following groups: (1) twice-daily low load resistance training with blood flow restriction (LL-BFR) or (2) heavier load resistance training (HL-RT) 3-days per week. Both treatment arms will be delivered alongside a standardised 3-week residential rehabilitation programme. Study outcomes will be recorded on course admission, course discharge and at three-months follow course, with the primary outcome time point being course discharge. The study design is outlined in Fig. 1.

The study protocol complies with the Standard Protocol Items Recommendations for Interventional Trials (SPIRIT) statement [60] and its checklist is included as supplementary material. The study has been approved by the Army Scientific Advisory Committee (SAC) and Ministry of Defence (MOD) research ethics committee (2129/MoDREC/2022) and is registered with ClinicalTrials.org (Trial registration number: NCT05719922). The study sponsor is the Head of Research and Clinical Innovation within UK Defence Medical Services. The study is joint funded through the Defence Medical Services Research Steering Group (DMSRSG), University of Bath and Versus Arthritis Centre for Sport and Exercise Osteoarthritis Research.

### Study setting

The study will be conducted at five RRUs across UK Defence Rehabilitation. Each RRU delivers a standardised 3-week residential rehabilitation course for serving personnel with MSKI. The exercise rehabilitation component of each course is led by a physiotherapist and exercise rehabilitation instructor (ERI). Details of course components are outlined in the supplementary material 1. The two treatment group interventions will be delivered alongside this standardised rehabilitation programme.

### Participants, recruitment, and screening

Prior patient and public engagement with all relevant stakeholders within UK Defence Rehabilitation to discuss strategies for achieving adequate participant enrolment to reach target sample size was conducted. All patients referred to an RRU assessment clinic with persistent knee pain will be screened against the eligibility criteria by a sport and exercise (SEM) medicine physician and/or senior physiotherapist.

#### Inclusion criteria

Patients will be eligible for the study if they have mechanical knee pain for at least three months; present with clinical signs and symptoms of knee pain arising from the tibiofemoral or patellofemoral joint diagnosed by SEM physician and/or senior physiotherapist; have reduced occupational employability medical grade secondary to their knee pain; report progression of resistance training load within the patient’s rehabilitation programme is limited by knee pain; aged between 18 and 55 years and are available to attend for the entire duration of the RRU course and a review appointment three-months following course.

#### Exclusion criteria

Patients will be ineligible for the study if they present with any medical contraindication related to LL-BFR (Table 1); diagnosed tibial, femoral or patella fracture and/or dislocation; present with instability in the knee resulting from ligament deficiency; present with clinical signs and symptoms of patellar tendinopathy; have planned surgery over the study period; restricted knee range of movement; clinical signs and symptoms of non-musculoskeletal or serious pathological condition (i.e. Inflammatory arthropathy, infection or tumour) or referred pain from non-local pain source; present with any physical impairment or co-morbidities (including cardio-vascular disease) precluding the safe participation in the rehabilitation programme and/or assessment procedures; have received cortico-steroid or analgesic injection intervention within the previous 7-days or previous knee surgery within the last 12-months to the affected limb.

**Table 1:** Medical exclusion criteria

**Medical Exclusion criteria**
History of cardiovascular disease including hypertension, peripheral vascular disease, thrombosis/embolism, ischaemic heart disease, myocardial infarction
History of the following musculoskeletal disorders: rheumatoid arthritis, avascular necrosis or osteonecrosis, severe osteoarthritis
History of the following neurological disorders: Peripheral neuropathy, Alzheimer’s disease, amyotrophic lateral sclerosis, Parkinson’s disease, severe traumatic brain injury
Varicose veins in the lower limb
Acute viral or bacterial upper or lower respiratory infection at screening
Known or suspected lower limb chronic exertional compartment syndrome (CECS)
Postsurgical swelling
Pregnant or medically downgraded post-partum
Surgical insertion of metal components at the position of cuff inflation
History of any of the following conditions or disorders not previously listed: diabetes, active cancer
History of elevated risk of unexplained fainting or dizzy spells during physical activity/exercise that causes loss of balance
History of haemorrhagic stroke or exercise induced rhabdomyolysis

Eligible participants will be informed of the study objectives, procedures, and interventions in both written and verbal format. Those expressing an interest to participate will be contacted by a member of the research team inviting them to participate in the clinical RCT. Participants meeting the eligibility criteria, who have read and understood the participant information sheet and volunteer to participate in the study, will be emailed an e-consent form to sign electronically. Participants who provide informed consent will be invited to participate in the nested mechanistic study. Those who volunteer for the nested mechanistic study will provide a separate written informed consent upon arrival at Defence Medical Rehabilitation Centre (DMRC), Stanford Hall on their first assessment day.

#### Randomisation

A permuted block randomisation method with a 1:1 ratio of the two treatment arms will be used using random block sizes. Randomisation will be stratified by study site, biological sex at birth, and diagnostic sub-group. A plain language statement will inform participants that they have an equal chance of receiving the HL-RT or LL-BFR intervention. A concealed list will be used to assign group allocation, this will be performed by an independent administrator not involved in the recruitment, treatment, or assessment of study outcomes.

### Intervention

Both groups will receive standardised rehabilitation care at their regional unit as per the course programme (supplementary file 1). The only component of treatment that differs between the two groups is the RT intervention prescribed for the quadriceps-based resistance exercise. Participants will be randomised into one of two RT groups. Group 1 will perform 1 set of 30 repetitions followed by 3 sets of 15 repetitions of LL-BFR at 20% 1-RM twice daily. Group 2 will perform 4 sets of 12 repetitions of HL-RT at ~ 70% 1-RM without BFR; aligned with conventional rehabilitation practice. Primary RT methods for the strength development of the quadriceps muscles will consist of two lower-limb exercises (1) unilateral leg press using a leg press machine, (2) unilateral knee extensions using a knee extension machine or ankle weights when clinically indicated. Resistance exercise order will consist of leg press followed by knee extension.

#### Low load resistance training with blood flow restriction (LL-BFR)

The blood flow restriction cuff will be securely fitted to the most proximal portion of the thigh by the exercise therapist. Prior to exercise, a personalised tourniquet pressure (PTP) of 80% of limb occlusion pressure will be determined, using the automated features inbuilt to the blood flow restriction (BFR) system (Delfi Medical, Vancouver, Canada). Participants will be asked to perform 1 set of 30 repetitions, followed by 3 sets of 15 reps of each exercise at 20% of their predicted 1-RM with an inter-set interval of 30 s of each exercise whilst wearing the BFR cuff. Between exercises the cuff will be deflated for 3 min. Exercises will be performed using a 1:0:1 tempo (1 s concentric phase; no pause; and 1 s eccentric phase). Training will be performed twice daily separated by interludes of at least 5 h.

#### Heavier load resistance training (HL-RT)

Participants will be asked to perform 4 sets of 12 repetitions for the leg press and knee extension exercise at 70% of their predicted 1-RM with an inter-set interval of 2 min. This sequence of exercise will be repeated 3 times per week during the 3-week rehabilitation course. Whilst the HL-RT protocol closely matches the total work volume of LL-BFR over the 3-week period, it is recognised that the treatment arms are not matched in other training parameters such as frequency, intensity, session volume, or rest periods. However, this exercise prescription was identified to reflect, as best, the current RT recommendations in rehabilitation [4].

### Main RCT outcome measures

Measurements will be assessed at course admission (T1), course discharge (T2) and at 12-weeks following course (T3), with the primary outcome time point being course discharge (T2). See Table 2 for schedule of data collection. The following subheadings include test overviews and justifications, whilst test procedures are outlined in supplementary file 2.

**Table 2:** Schedule for data collection

**Domain/Outcome Measure**	**T1**	**T2**	**T3**
**Baseline Assessment**			
Demographics Questionnaire	X		
Health Anxiety Depression Scale (HADS)	X		
**Patient Reported Outcomes**			
*Function*			
Lower Extremity Functional Scale (LEFS)*	X	X	X
Knee Injury and Osteoarthritis Outcome Score (KOOS)	X	X	X
Musculoskeletal Health Questionnaire (MSKHQ)	X	X	X
Patient Specific Functional Scale	X	X	X
Physical Activity Questionnaire	X		X
*Pain*			
Numeric Pain Rating Scale (NPRS)	X	X	X
*Psychosocial*			
Tampa Scale of Kinesiophobia (TSK)	X	X	X
Sports Injury Rehabilitation Beliefs Survey (SIRBS)	X	X	X
*Occupation*			
Functional Activity Assessment (FAA)	X		X
**Physical Capacity Assessment**			
*Muscular performance*			
5-RM Lower-Limb Strength	X	X	X
Isometric Muscle Strength of hip and knee	X	X	X
Single Leg Heel Raises to Fatigue	X	X	X
*Movement pattern analysis*			
Qualitative Assessment of Single Leg Squat (QASLS)	X	X	X
*Pain provoking task performance*			
Decline Knee Bend	X	X	X
**Muscle Imaging**			
*Magnetic resonance imaging (MRI)*			
Anatomical Cross-sectional area (ACSA)	X	X	X
Muscle volume	X	X	X
*Ultrasound imaging*			
Fascicle length (F_L_)	X	X	X
Pennation angle (θp)	X	X	X
*MRI* + *ultrasound*			
Physiological cross-sectional area (PCSA)	X	X	X
**Neuromuscular Performance**			
*Isometric strength testing*			
Isometric mid-thigh pull	X	X	X
Maximum voluntary isometric contraction (MVIC)	X	X	X
*Kinetic and kinematic analysis of functional movements*			
Bilateral squat	X	X	X
Unilateral squat	X	X	X
Countermovement jump (CMJ)	X	X	X
*Isometric strength testing* + *ballistic task performance*			
Dynamic Strength Index (DSI)	X	X	X
**Blood sampling**			
Muscle damage	X	X	X
Inflammation	X	X	X
Oxidative stress	X	X	X
Endothelial function	X	X	X

#### Baseline characteristics

Baseline characteristics (T1) will include a participant demographics questionnaire and Health Anxiety Depression Scale (HADS). Personal and demographic characteristics including age, body height, body mass, body mass index (BMI), duration of symptoms, previous injuries, previous treatment, military occupation, duration of military service, smoking and drinking habits.

#### Patient reported outcome measures

The Lower Extremity Functional Scale (LEFS) at course discharge (T2) will be the primary outcome measure. The LEFS is a patient-reported outcome measure (PROM) that measures functional status in patients with lower limb MSKI [61]. Ability to perform 20 activities, ranging from walking to running on uneven ground, are self-reported from 0–4 with higher scores indicating better function. The minimum clinically important difference (MCID) of the LEFS is 9 points. The LEFS is a validated tool and has demonstrated good test-retest reliability and responsiveness in individuals with persistent knee pain [61–63].

Secondary patient reported outcome measures will record patient reported levels of function, pain, fear of movement, rehabilitation beliefs, physical activity and occupational status. Questionnaires include: the Knee Injury and Osteoarthritis Outcome Score (KOOS) [64], Patient Specific Functional Scale [65], Musculoskeletal Health Questionnaire (MSKHQ) [66], Tampa Scale of Kinesiophobia (TSK) [67], Numeric Pain Rating Scale (NPRS) [68], Functional Activity Assessment (FAA) [69], Sports Injury Rehabilitation Beliefs Survey (SIRBS) [70] and the International Physical Activity Questionnaire (IPAQ). Questionnaires will be distributed at T1, T2 and T3 via email using a research electronic data collection application (REDCap).

#### Physical/functional capacity tests

Physical/Functional capacity tests will assess muscle strength at the hip and knee, calf muscular endurance, single leg movement patterning and pain-free maximum loaded knee flexion. Tests will be conducted and recorded by the study site clinicians. All assessments include testing procedures that can be applied in the clinical setting.

##### 5-RM leg press

Unilateral muscle strength will be assessed using a dynamic 5-RM test, defined as the maximal load (kg) that the participant can lift five times consecutively with the correct lifting technique. This will be performed on a leg press machine to assess multi-joint functional strength and is aligned with current clinical care practice. Multiple repetition strength assessments expose the skeletal muscles, connective tissue and joints to lower loads when compared to maximal strength testing, such as 1-RM testing, and are associated with a lower risk of injury and symptoms of delayed muscle soreness [71]. Thus, 5-RM testing is considered a more suitable assessment protocol for injured personnel in the rehabilitation setting. This test has demonstrated good test-retest reliability and can be used as a valid predictor of maximal strength (1-RM) [72, 73].

##### Isometric hip and knee strength

**Fig. 2:**
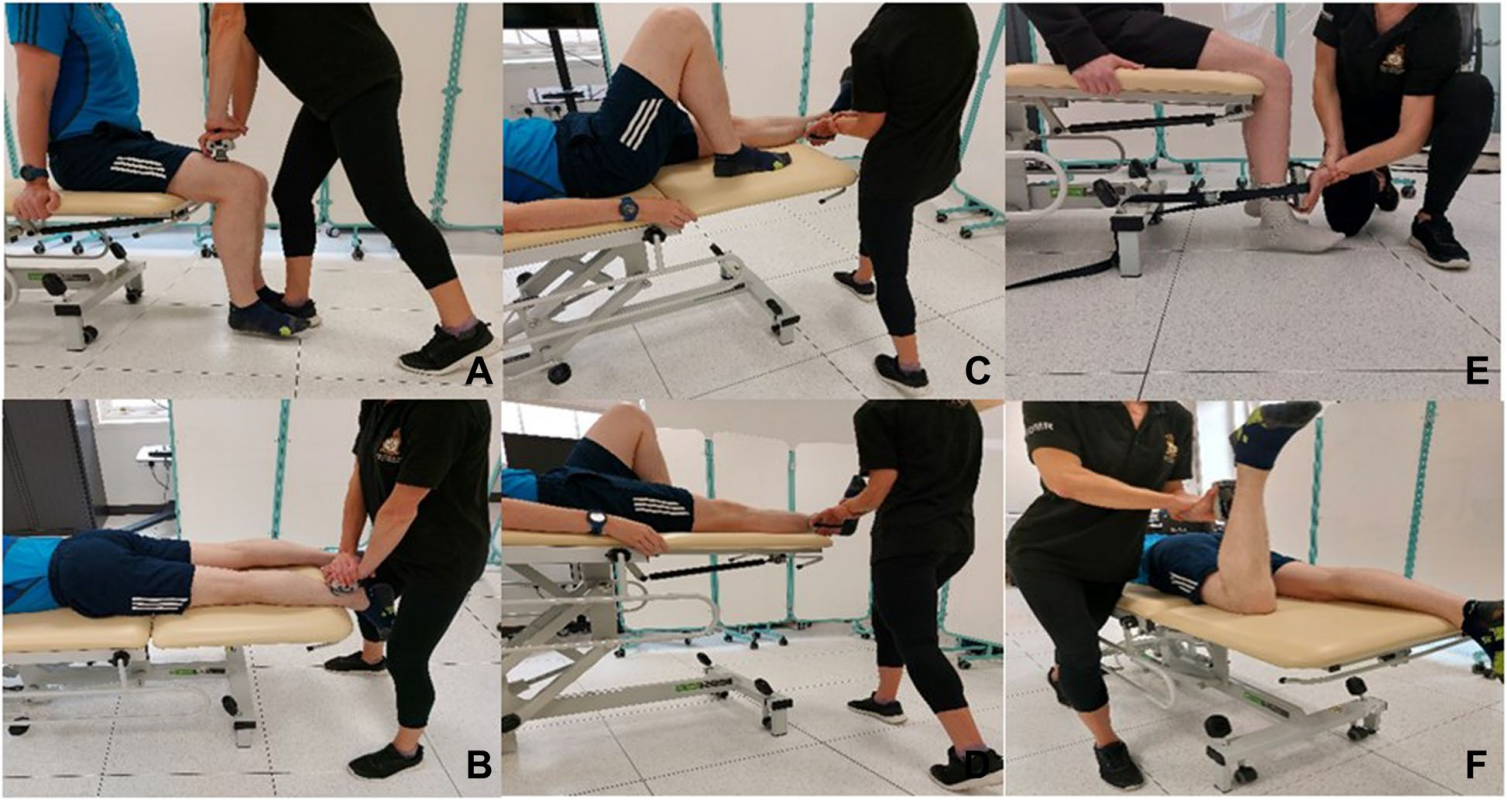
Testing positions and order for isometric strength assessment. **A** Hip flexors **B** Hip extensors **C** Hip adductors **D** Hip abductors **E** Knee extensors **F** Knee flexors

Isometric muscle strength will be assessed at the hip and knee in frontal and sagittal planes. This will provide an indication of muscular adaptation at the knee but also muscles proximal to the cuff [74–76]. Measurements will be taken using a wireless digital Lafayette hand-held dynamometer (HHD) (Lafayette, Indiana, United States). Hand held dynamometry is considered a valid practical method to assess muscle isometric strength [77]. The isometric ‘make-test’ was chosen as isometric loading induces less stress on the musculoskeletal system than eccentric loading (‘break-test’), which is a key consideration when testing individuals with a physical injury [78, 79]. A standardised HHD measurement technique will use procedures often applied in the clinical setting (Fig. 2). The testing positions offer sufficient mechanical advantage for the testers and has demonstrated good to excellent interrater reliability (ICC 0.82–0.98) and low test-retest variation (< 10%) when measuring isometric lower limb muscle strength [80].

*Heel-raise test* The heel-raise test is used in clinical practice to assess properties of the calf muscle-tendon unit (MTU) [81, 82]. The heel-raise test involves repetitive concentric–eccentric muscle action of the plantar-flexors in a single leg stance and is quantified by the total number of raises performed. This test demonstrates good reliability and has traditionally been used to assess various calf MTU properties including muscular strength, endurance and fatigue [82–85]. It is acknowledged that the ankle plantar flexors (gastrocnemius and soleus muscles) will not be subjected to high contracting forces during the leg press and knee extension exercises and are therefore not under a comparable biological stimulus as the quadriceps muscle. However, it is important to consider the holistic impact of any new exercise rehabilitation protocol on physical metrics considered important to the population of interest. Due to the high demand placed on the ankle plantar flexors during military tasks, such as running, load carriage and multidirectional speed and agility, restoring calf capacity is a common goal during military specific rehabilitation programmes [86]. Therefore, it is of clinical interest to determine whether any muscular adaptation occurs in this functionally important muscle group.

**Fig. 3:**
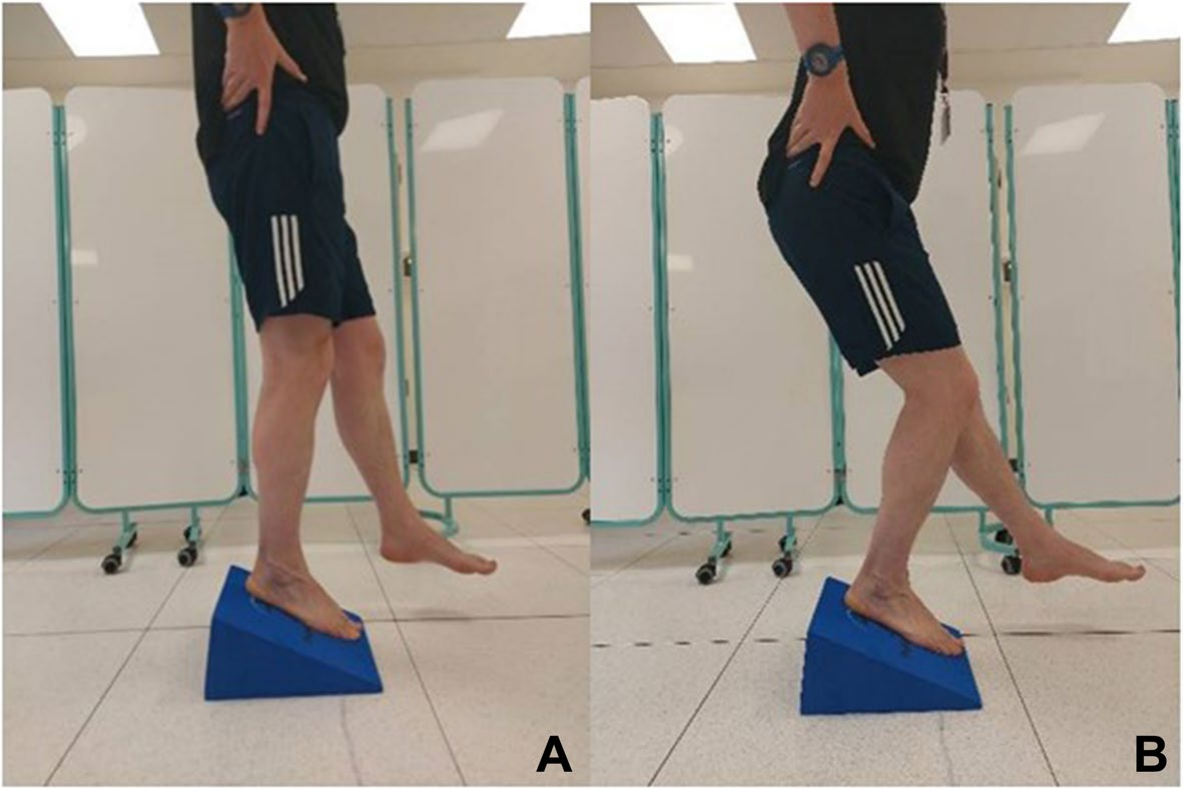
Decline knee bend task. **A** Start position; **B** End position

*Decline knee bend* Knee pain is commonly reported during specific weight-bearing knee flexion tasks such as stair climbing, running, jumping [87]. The decline step-down test is a clinical performance test that replicates these movement patterns and thus assesses changes in knee pain and function that is directly relevant to these commonly reported problems [88]. The maximum flexion angle achieved at the knee joint without increasing symptomatic knee pain will be determined using video analysis (RPC). A box with a 25º decline angle will be used to prevent ankle dorsi-flexion being the limiting factor during this task (Physio Foam, UK) (Fig. 3).

*Qualitative analysis of single leg squat (QASLS)* The qualitative analysis of single leg squat (QASLS) is a scoring system designed to identify segmental sub optimal behaviour following performance of a single leg squat [89]. This testing procedure will be video recorded and independently scored by two members of the research team (RPC and KML). The QASLS involves dichotomous scoring of the movement strategies occurring in individual body regions. Movement analysis is subdivided into six categories-arm strategy, trunk alignment, pelvic plane, thigh motion, knee position and steady stance. Pelvic plane, thigh motion, knee position and steady stance each have two performance points. This test has demonstrated very good to excellent inter and intra-rater reliability and good criterion validity against three-dimensional (3D) motion capture [89, 90].

#### Training load

A participant monitoring booklet (Table 3) will enable daily monitoring of training load, participant wellness scores, symptomatic knee pain and localised muscle discomfort during the 3-week rehabilitation course. This will be recorded in a participant booklet that is completed daily by the participant and therapist.

**Table 3:** Participant monitoring booklet

**Domain**	**Measure**	**Frequency**
Participant Wellness Score	Likert scale (0–5) of 5 dimensions	Once daily, AM
Training load	Sets, reps, load completed	Immediately post study intervention session
Session rate of perceived exertion (sRPE)	scale of 0 to 10	Immediately post study intervention session
Symptomatic knee pain	Visual analogue scale (VAS), 100 mm horizontal line	Immediately prior to starting the exercise, during the exercise and then 5 min post-exercise
Muscular discomfort	Visual analogue scale (VAS), 100 mm horizontal line	Immediately prior to starting the exercise, during the exercise and then 5 min post-exercise.
Symptomatic knee pain during a pain provoking functional task (single-leg knee bend)	Visual analogue scale (VAS), 100 mm horizontal line	Every third session for LL-BFR group
		Every session for HL-RT session.

#### Muscular discomfort and symptomatic knee pain

A visual analogue scale (VAS) will be used to measure pain intensity. The VAS uses a 100 mm horizontal line anchored by the terms ‘no pain’ (0) and ‘worst possible pain’ (100). The VAS response format has shown good internal consistency, is easy to understand, is in wide clinical use, and has been sufficiently evaluated in clinical trials [91]. Levels of muscular discomfort and symptomatic knee pain will be recorded immediately prior to starting the quadriceps-based exercise, during the exercise and then 5 min post-exercise. Previous studies have demonstrated a hypoalgesia response up to 45 min post BFR training [49]. Therefore, all participants will also be asked to score symptomatic knee pain during a pain provoking functional task (single-leg knee bend) immediately prior and 15 min following the cessation of exercise. These pain-related outcome measures will be repeated at the start, middle and end of each treatment week to monitor how pain response changes over time to both intervention arms. Muscular discomfort and symptomatic knee pain will be monitored during the 3-week rehabilitation admission using a participant monitoring tool (Table 3).

### Nested mechanistic study outcome measures

All mechanistic based outcome measures will be assessed at DMRC Stanford Hall 3-days prior to T1 (course admission), 3-days following T2 (course discharge) and at T3 (three-months following discharge). T2 data will be collected 5-days following the final exercise session. This is to provide sufficient recovery for maximal torque generation following resistance exercise and for the effects of exercise induced muscle swelling to subside [52, 92–95]. Outcome measures will be collected in the following order: blood sample, muscle imaging, bilateral squat, unilateral squat, countermovement jump (CMJ), isometric mid-thigh pull (IMTP), maximum isometric voluntary contraction (MVIC). Performing muscle imaging prior to neuromuscular testing ensures that exercise does not influence muscle morphology or architecture. The sequence of neuromuscular testing ensures that movements that place the greatest loading demands on the knee joint are scheduled towards the end of the testing session, reducing the probability of symptomatic pain or neuromuscular fatigue affecting subsequent task performance. Outcome data will be collected at the same time of day to minimise the effects of circadian rhythm on performance.

#### Muscle imaging

Participants will rest quietly in a supine position for 20 min prior to muscle imaging to allow for postural related fluid shifts to occur [96].

##### Anatomical cross-sectional area and volume

Anatomical cross-sectional area (ACSA) (cm^2^) and volume (cm^3^) of the involved thigh musculature will be measured using magnetic resonance imaging (MRI) with a Philips Ingenia Elition 3.0T X (Philips Healthcare, Best, The Netherlands). A T1-weighted, spin echo, axial plane sequence will be obtained with contiguous transverse images from the greater trochanter to the lateral condyle of the femur with a 1.0 cm slice thickness and no inter-slice gap. The MRI data will be anonymised and transferred onto a study laptop for analysis using a public domain image analysis software (Image J, v1. 48, National Institutes of Health, Bethesda, USA). For each slice, ACSA and volume will be calculated for each muscle within the quadriceps femoris and for the global hamstring compartment. Inter- and intra-rater reliability will be calculated from the repeated analysis of the first five MRI scans.

##### Quadriceps muscle architecture

Pennation angle (θp) and fascicle length (F_L_) of the vastus lateralis (VL) muscle on the involved leg will be measured using a B-Mode Philips Epiq 5G ultrasound device and a 5 cm Philips eL18-4 PureWave transducer (Philips Healthcare, Best, The Netherlands). Measurements will be taken at 35, 50 and 65% of the distance between the lateral condyle of the femur and the greater trochanter, as muscle architecture is known to vary across the VL [97]. The knee will be flexed to 40º, as this is thought to represent the resting length of the VL, thereby minimising fascicle stretch or slackness [98–100]. Increases in θp are thought to represent an increase in sarcomeres in parallel and allows short muscle fibres to be packed within a limited volume, thus increasing force generation capacity [101, 102]. The acute angle between a fascicle and the deep aponeurosis will be defined as θp. The F_L_ has been proposed to reflect muscle fibre length and therefore the number of sarcomeres in series, which dictates the maximal shortening velocity of a muscle [39]. Measures of F_L_ will be obtained by tracing over a fascicle that extends from the superficial to the deep aponeurosis.

Ultrasound data will be anonymised and transferred onto a study laptop before being digitised using a public domain image analysis software (Image J, v1. 48, National Institutes of Health, Bethesda, USA). During digitisation, measurements at each ultrasound location will be performed on three different fascicles, with digitisation repeated three times. Mean VL θp and F_L_ values will then be used for statistical analyses. To ensure the same fascicles are measured at each time point, pre- and post-intervention images will be compared during analysis, with identifiable landmarks such as fat and connective tissue used as reference points [97, 103]. One researcher (KL) will perform all ultrasound assessments and will be trained by a consultant radiologist (ES) at DMRC over three, 1-h sessions. The first five participants will be assessed by both the researcher and the consultant radiologist to calculate intra- and inter-rater reliability.

##### Physiological cross-sectional area

Physiological cross-sectional area (PCSA) is proposed to represent the sum of the cross-sectional areas of all muscle fibres within a given muscle and is known to be the only architectural parameter which directly relates to maximal isometric force production [39]. An in vivo estimate of VL PCSA will be calculated using Eq. 1 [39, 104].

1 $$\mathrm{PCSA }\left({\mathrm{cm}}^{2}\right)=\frac{\mathrm{VL\, volume }\,({\mathrm{cm}}^{3})}{{\mathrm{F}}_{L} (cm)}$$

#### Neuromuscular performance

For each neuromuscular task, participants will be asked to rate their symptomatic knee pain before, during, immediately after and 5 min following each task using a VAS. Surface electromyography (sEMG) will be used during all neuromuscular testing using a wireless sEMG system (Trigno; Delsys Inc., Boston, MA). Standard Trigno, single differential sensors (Delsys Inc., Boston, MA) with a fixed 1 cm inter-electrode distance will be attached to the superficial quadriceps (vastus medialis, rectus femoris and VL), hamstrings (long head biceps femoris and semitendinosus). Electrodes will be attached on both legs and in the presumed orientation of the underlying fibres. Electrode locations will be prepared and defined in accordance with Seniam guidelines [105]. To ensure the reliability of electrode location between time points, the distance of each electrode relative to the two anatomical landmarks used to determine electrode location (as defined by Seniam) will be recorded at T1 and replicated at T2 and T3. Electrode orientation will also be measured at T1 (using a goniometer) and replicated at T2 and T3.

##### Isometric strength testing

Where pain is the primary limiting factor to performance, isometric strength testing may eliminate painful joint movement under loaded conditions and may provide a safer alternative for the quantification of force production [106–108].

The IMTP has demonstrated good-to-excellent reliability in measuring maximal strength [109], is currently implemented as a role fitness test within the British Army Physical Employment Standards and has previously been integrated into lower-limb rehabilitation settings within UK Defence rehabilitation [108]. The IMTP is a test that can assess multiple derivatives of maximal lower-limb muscle force production capabilities, including peak force, rate of force development and limb symmetry. The IMTP will be delivered using a previously established, standardised testing procedure [107] on a pair of portable force plates (Hawkin Dynamics, Portland, Maine, United States) located on the base plate of a mid-thigh pull rig (Absolute Performance, Cardiff, UK). Force-time data will be sampled at 1,000 Hz and will be visually assessed against a previously established criteria, with invalid trials repeated [107].

Knee extension and knee flexion MVIC’s will be performed using an isokinetic dynamometer (Biodex Multi-Joint system Pro, Biodex Medical Systems Inc, Shirley, New York). The use of isokinetic dynamometry is widely considered to be the gold standard for measuring muscle torque [110]. All isokinetic dynamometry will be conducted with participants at a hip angle of 90°, with knee angles at 60° and 45° for knee extension and flexion respectively; these joint angles are known to produce maximal torque values and electromyographic amplitudes and have previously been performed safely by individuals with pain arising from the patellofemoral and tibiofemoral joints [111, 112].

##### Kinetic and kinematic analysis during functional movements

Three-dimensional motion capture and force plate data will be collected for the analysis of kinetic and kinematic variables during the bilateral squat, single leg squat and the CMJ using a 20-camera 3D system (Vicon MX system, Oxford Metrics, Oxford, England) and two AMTI force plates (Boston, USA). Data will be captured at 200 Hz and 2,000 Hz for motion capture and force plate data respectively. Forty-six retroreflective markers will be placed on the skin over anatomical landmarks by the same researcher (KML), establishing an eight-segment model including the foot, shank, thigh, pelvis and trunk [113]. Data will be labelled in Vicon Nexus (Oxford Metrics, version 2.12, Oxford, England) before being processed in Visual 3D (C-motion, version 2022.08.3, Rochelle, USA).

The performance of bilateral and unilateral squats allows for the detailed assessment of clinically relevant kinetic and kinematic variables during functional tasks commonly used during patient clinical assessments [114]. Five trials will be performed, where participants will be asked to squat to a depth beyond 60° and return to an upright position over a 4 s metronome paced cycle. Data from the first and fifth trial will be discarded, with mean values from the middle three trials used for analysis.

The CMJ can yield valuable insight into an individual’s neuromuscular function, ballistic force production and stretch-shortening (SSC) capabilities [115]. The CMJ has been demonstrated to be a valid and reliable measure of lower-body explosive strength [116] and has strong, positive associations with occupational performance in military settings [117]. Three trials will be performed, with mean values used for analysis. No coaching on technique will be offered during any movement, as technique cueing is known to alter kinetic and kinematic variables and may therefore encourage participants to adopt movement strategies which do not represent their typical movement patterns [118]. On the contrary, self-selected movement patterns possess high correlations with the joint loading encountered during daily living and occupational tasks [114].

##### Dynamic strength index

The dynamic strength index (DSI) provides a ratio of the force that an individual can produce during isometric and ballistic tasks and is thought to provide insight into an individual’s training status by highlighting performance deficits [119]. A DSI will be calculated using peak force values from the IMTP and the propulsion phase of the CMJ and is shown in Eq. 2. These tasks have previously been demonstrated to produce reliable DSI data [120].

2 $$\mathrm{DSI}=\frac{\mathrm{CMJ\, Peak\, Force }(\mathrm{N})}{\mathrm{IMTP\, Peak\, Force }(N)}$$

#### Blood sampling

A 20 ml venous blood sample will be obtained from an antecubital vein to assess the chronic effects of LL-BFR and HL-RT on markers of muscle damage, inflammation, and endothelial function. Samples will be obtained at the same time of day (07:00–10:00 h), following a ≥ 12 h fast, with abstinence from alcohol (≥ 24 h), caffeine (≥ 12 h) and vigorous exercise (72 h). These measurement procedures are designed to minimise pre-analytical variability in biomarkers [121].

### Blinding

Given the nature of BFR, it is not possible to blind participants to their treatment allocation. The clinical staff who deliver the study interventions and collect outcome data for the main RCT must also be, by necessity, un-blinded. The following outcome measures will be assessor blinded: Decline knee bend, QASLS and all the outcome measures collected in the nested mechanistic study.

### Study site training

Prior to data collection, all study site clinicians will receive BFR specific training from a recognized external provider. Training on the recruitment, intervention delivery and data collection procedures will be provided by the principal investigators (RPC and KML) and the chief investigator (PL). Each study site will have a named lead study practitioner (a physiotherapist or exercise rehabilitation instructor) who will take responsibility for day-to-day management of the trial at their respective rehabilitation unit. In addition, the research team will audit and conduct interim research training days for each study site to allow training updates.

### Sample size

#### Main randomised control trial

A sample size of 150 participants (75 in each arm) will be recruited into the study. The study is powered to detect a minimum clinically important difference (MCID) in the Lower Extremity Functional Scale (LEFS) using a between group MCID of 9 points and an estimated standard deviation of 15.8 [61]. From this an effect size, f, of 0.285 was calculated. The expected sample size for 0.80 power to detect an effect size (f) of 0.285 at a level of significance α = 0.05 is 122 participants (61 In each group). By estimating an approximate 20% dropout rate we concluded a sample size of 150, with 75 in each intervention arm. The G Power 3.1.9.7 software was used to calculate the sample size. This sample size is also sufficient to detect a MCID of 8 points in the KOOS ADL subscale with a statistical significance of *α* = 0.05 and power of > 0.80 where the MCID is 8 points and expected standard deviation is 8.9 [122, 123].

#### Nested mechanistic study

The nested study will recruit a sub-sample of participants from the main RCT. No group x time interaction is hypothesised to occur for the primary dependent variable of quadriceps femoris muscle volume. Therefore, a within factors priori power analysis was performed to calculate the sample size required to detect a within group change in quadriceps femoris muscle volume between T1 and T2. Based on data from the ADAPT pilot study [26], which reported an effect size of *d* = 0.35, the minimum sample size required is *n* = 52. By estimating an approximate 20% dropout rate we concluded a sample size of *n* = 64 (*n* = 32 in each intervention arm) is required.

### Statistical methods and analysis

Descriptive data will be reported as the mean and standard deviation (SD) for continuous variables and frequency statistics for non-continuous variables. Prior to statistical analysis, tests to establish the normality of data distributions will be performed and, where appropriate, variance-stabilising transformations will be applied. Analysis will be conducted on a modified intention to treat basis, including all available outcomes at course discharge regardless of compliance to treatment. All tests will be two-sided, and alpha will be set at 0.05. Assumptions for all tests will be considered and adhered to. In the primary analysis, a mixed-effects regression analysis will be used to assess the effects of the interventions on primary outcome scores at course discharge (T2) between the two treatment groups, after adjusting for fixed effects of biological sex at birth and baseline (T1) primary outcome scores, with study site centre included as a random effect. The treatment effect estimates will be presented from the adjusted mixed model with 95% confidence intervals (CI) and P-values. This mixed-effects model will be used for all secondary outcome measures. Supporting analysis of the primary outcome will include: A per-protocol analysis including patients with compliance > 80% to intervention; a repeat of the primary analysis with additional adjustments for baseline values and a mixed effects model to compare primary outcomes at three-months post intervention between the two treatment groups. In addition to the primary adjusted analysis, the unadjusted mean differences between groups will be compared using t-test for independent groups, reporting 95% CI. Sub-group analysis will be conducted by diagnostic sub-group, biological sex at birth, age and pain levels at baseline. Study participant flow will be recorded and reported in accordance with the Consolidated Standard of Reporting Trials (CONSORT) guidelines. Analysis will be conducted on a pairwise case basis. Therefore, all missing data will be reported, and patterns investigated. Sensitivity analysis will be conducted using multiple imputation techniques to assess the effect of missing data on primary outcomes. Post intervention adverse events between groups using Fisher’s exact test. All analysis will be conducted using SPSS.

At *n* = 20 an interim analysis of study fidelity and reliability of data recorded will be performed. At *n* = 50 and *n* = 100 additional interim analysis will be performed to establish any clear superiority of one intervention over another. If the magnitude of the difference warrants consideration for stopping the trial, this will be discussed with the chair of the MOD ethics committee.

### Data management

All data will be entered into REDCap by study site clinicians or participants directly. Participants will be identified through a unique identification key. Data access will be restricted to pre-identified clinicians at each individual study site to ensure confidentiality. Only the research team involved in data analysis will have data exportation rights. Participant monitoring booklets will be stored securely at each study site and collected periodically by the research team. All anonymised data and data dictionary will be retained, both raw and processed, since both may be useful in future studies. As data used in the course of this project is crown copyright protected. On completion of the study raw and processed data underpinning publications will be archived and stored securely on the electronic data archiving system at the Academic Department of Military Rehabilitation (ADMR) within the MOD. Data will be retained for 10-years. This trial is embedded within an existing clinical care pathway and as it is not testing new pharmaceutical products or drugs a formal data monitoring committee was not required. However, a study steering group (RPC, KML RJC and PL) will meet weekly to discuss matters arising from each study site location related to adherence and data management.

### Adverse events

Information on any unexpected adverse events deemed to be related to study participation will be collected and reported to the chief investigator within 24-h of its occurrence. A standardised proforma will be completed by the study site clinician which will detail the time and date of the incident, severity of the event, the relationship to the study and the following action taken and outcome. All serious adverse events will be recorded and discussed directly with the MODREC. Reporting of safety incidents will be duplicated using existing clinical health and safety reporting procedures and in accordance with the principles of good clinical practice (GCP).

## Discussion

The use of LL-BFR in rehabilitation is increasing. Previous studies have highlighted the benefits of this approach regarding muscular strength, hypertrophy, function and pain modulation within different knee pathologies [45–49]. Other study protocols typically deliver LL-BFR 2–3 times a week over a 6–8-week duration, mirroring a traditional resistance training programme approach. However, one of the advantages of LL-BFR is providing an exercise stimulus in the absence of high mechanical stress. This enables high frequency LL-BFR to be used over short durations not deemed feasible with heavy load resistance training protocols. In healthy adult populations, high frequency (twice daily) LL-BFR has shown to be a safe and effective form of exercise intervention that can elicit favourable changes in lower limb muscle strength and muscle CSA over a 2–3 week duration [54–58]. However, the potential benefits of replicating this intensive approach in the rehabilitation setting when compared to conventional rehabilitation are still unclear. This study aims to expand on the pilot and feasibility RCT delivered in UK Defence rehabilitation in 2018 [26] into a comprehensive, fully powered, pan-UK Defence multi-centre RCT. This will help determine the effectiveness and clinical utility of this approach when utilised in an intensive rehabilitation setting.

The effects LL-BFR immediately following a course of treatment are well documented. However, less is known about the longer-term impact on an individual’s rehabilitation pathway. Therefore, this study protocol includes a follow-up assessment at three-months following intervention, this will provide some additional insight as to whether any changes in outcome seen immediately post intervention led to a meaningful lasting impact for that participant and their recovery.

To our knowledge, this is the first study to assess the effect of LL-BFR on neuromuscular performance and biomechanical adaptations in a military cohort with persistent knee pain. It will also be the first study to determine whether LL-BFR elicits an increase in agonist PCSA. The clinical implications of these findings are that LL-BFR is a rehabilitation modality that has the potential to induce positive clinical adaptations in the absence of high mechanical loads and therefore could be considered a treatment option for patients suffering significant functional deficits who are unable to tolerate heavy load RT. This study will aim to optimise rehabilitation outcomes and improve the time-and cost-effectiveness of the service delivered across UK Defence Rehabilitation and beyond. Results will provide insight and knowledge to the clinical and scientific community to not only those embedded within Defence Rehabilitation, but also those working in civilian sector organisations and professional sport in the UK and abroad.

## Supplementary Information

**Additional file 1.** ADAPTation to therapeutic resistance training (ADAPT) Study Intervention Guide.

**Additional file 2.** ADAPT Study Outcome Measures.

**Additional file 3.** ADAPT Study Eligibility Criteria.

**Additional file 4.** SPIRIT-Outcomes 2022 Checklist (for combined completion of SPIRIT 2013 and SPIRIT-Outcomes 2022 items)^a^.

## Data Availability

Data sharing is not applicable to this article as no datasets were generated or analysed during the current study.

## Abbreviations

ACSA: Anatomical Cross-Sectional Area
ADMR: Academic Department of Military Rehabilitation
BFR: Blood Flow Restriction
BMI: Body Mass Index
CI: Confidence Intervals
CK: Creatine Kinase
CMJ: Countermovement Jump
CONSORT: Consolidated Standard of Reporting Trials
CSA: Cross-Sectional Area
DMRC: Defence Medical Rehabilitation Centre
DMSRSG: Defence Medical Services Research Strategy Group
DOMS: Delayed Onset Muscle Soreness
DSI: Dynamic Strength Index
EIMD: Exercise Induce Muscle Damage
ERI: Exercise Rehabilitation Instructor
FAA: Functional Activity Assessment
F_L_: Fascicle Length
GCP: Good Clinical Practice
HADS: Health Anxiety Depression Scale
HHD: Hand-Held Dynamometer
HL-RT: Heavier Load Resistance Training
IMTP: Isometric Mid-Thigh Pull
IPAQ: International Physical Activity Questionnaire
KOOS: Knee Injury and Osteoarthritis Outcome Score
LEFS: Lower Extremity Functional Scale
LL-BFR: Low Load Blood Flow Restriction
LOP: Limb Occlusion Pressure
MCID: Minimum Clinically Important Difference
MOD: Ministry of Defence
MRI: Magnetic Resonance Imaging
MSKHQ: Musculoskeletal Health Questionnaire
MSKI: Musculoskeletal Injury
MTU: Muscle-Tendon Unit
MVIC: Maximum Isometric Voluntary Contraction
NPRS: Numeric Pain Rating Scale
PCSA: Physiological Cross-Sectional Area
PROM: Patient-Reported Outcome Measure
PSFS: Patient Specific Functional Scale
PTP: Personalised Tourniquet Pressure
QASLS: Qualitative Analysis of Single Leg Squat
RCT: Randomised Controlled Trial
REDCap: Research Electronic Data Collection Application
RM: Repetition Maximum
ROM: Range of Movement
RRU: Regional Rehabilitation Unit
RT: Resistance Training
SAC: Scientific Advisory Committee
SD: Standard Deviation
SEM: Sport and Exercise Medicine
sEMG: Surface Electromyography
SIRBS: Sports Injury Rehabilitation Beliefs Survey
SPIRIT: Standardised Protocol Items Recommendations for Interventions Trials
SSC: Stretch-Shortening Capabilities
TSK: Tampa Scale of Kinesiophobia
VAS: Visual analogue scale
VL: Vastus Lateralis
θp: Pennation Angle
3D: Three-Dimensional
