# Supplementary material for: ADAPTations to low load blood flow restriction exercise versus conventional heavier load resistance exercise in UK military personnel with persistent knee pain: protocol for the ADAPT study, a multi-centre randomized controlled trial
Source: BMC Musculoskelet Disord. 2023 Jul 17;24:580. doi: 10.1186/s12891-023-06693-3 (PMC10351180; doi:10.1186/s12891-023-06693-3)
Supplement: Supplementary file 1 — Additional file 1. ADAPTation to therapeutic resistance training (ADAPT) Study Intervention Guide. [file 12891_2023_6693_MOESM1_ESM.pdf]

Defence Medical
Rehabilitation
Centre
Stanford Hall

ADAPTation to therapeutic resistance training (ADAPT) Study

Intervention Guide

INTRODUCTION

This guide is intended to provide supervising clinicians with direction for the interventions employed in the ADAPT research trial. The intervention guide adheres to standards outlined in the Template for Intervention Description and Replication (TIDieR) and Consensus on Exercise Reporting Template (CERT) which sit as extensions of the Consolidated Standards of Reporting Trials (CONSORT) statements for reporting randomised controlled trials (1, 2). The guide is based on the available published evidence and clinical practice guidelines recommendations for the conservative treatment of persistent knee pain (both patellofemoral and tibiofemoral pain).

This intervention guide covers:

a. **Section 1: Delivery of the resistance training protocol**. This will describe the two resistance training protocols for the two intervention arms of the study that is conducted whilst the participant attends the residential rehabilitation course.
b. **Section 2: Residential rehabilitation course.** This section details the overarching components of the residential rehabilitation courses delivered to participants in both the intervention arms at the regional rehabilitation unit study sites.
c. **Section 3: Rehabilitation following course.** This section provides clinical guidelines for the 12-week individualised exercise programme that is prescribed for participants in both intervention arms to continue with following course.

SECTION 1: DELIVERY OF THE RESISTANCE TRAINING PROTOCOL

Both groups will receive conventional rehabilitation care at their regional unit. The only component of treatment that differs between the two groups is the resistance training (RT) intervention prescribed to the quadriceps-based resistance exercises. Participants will be randomised into one of two resistance training groups.

Group 1 will perform twice daily low-load (~20% 1-RM) resistance training with BFR (LL-BFR).

Group 2 will perform 4 sets of 12 repetitions of heavier load (~70% 1RM) resistance training (HL-RT) without blood flow restriction (BFR); aligned with more conventional rehabilitation training methods.

Primary RT methods for the strength development of the quadriceps muscles will consist of two lower-limb exercises: Unilateral leg press using a leg press machine and unilateral knee extensions using a knee extension machine or ankle weights when clinically indicated (3, 4). Resistance exercise order will consist of performing the leg press movement followed by knee extension. Prior to the resistance exercise, each participant will undergo a standardised 5-minute progressive warm-up on a stationary bike (Wattbike Ltd, Nottingham, UK).

Group 1: Low Load with Blood Flow Restriction (LL-BFR)

Participants will be asked to perform 4 sets of each exercise whilst wearing a blood flow restriction cuff on the proximal thigh of their affected limb with a personalised tourniquet pressure (PTP) set at 80% Limb Occlusion Pressure (LOP). PTP will be determined prior to each resistance training session (see below). Exercises will be performed with the following sequence of repetitions; 1 set of 30 reps followed by 3 sets of 15 reps (75 repetitions in total) at ~20% of 1RM, with an inter-set interval of 30 seconds, in accordance with previously published protocols (5, 6). Between exercises the cuff will be deflated for 3 minutes. A metronome will be set at 60 bpm, with 1s for the concentric phase; no pause; and 1s for the eccentric phase of the lift (1:0:1 tempo) to ensure consistency of lifting between patients. Training will be performed twice daily, in the morning (between 08:00-11:00) and afternoon (between 14:00-17:00) from Monday to Thursday and once on Friday morning (between 08:00-10:00). Daily RT sessions will always be separated by interludes of at least 5 hours. Over the 15 days of residential rehabilitation, clinical assessments will be carried out on the first day and final two days of the 3-week admission. This allows a maximum of 21 training sessions during the 3-week residential admission.

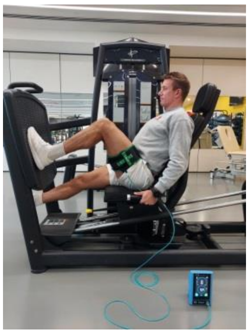

A.

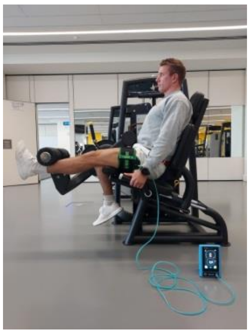

B.

Figure 1: Resistance exercises prescribed. A, Leg Press (with BFR); B = Knee-Extension (with BFR)[1]

Determining Personalised Tourniquet Pressure (PTP) for the LL-BFR Group

PTP will be set at 80% of Limb Occlusion Pressure (LOP) in accordance with current available best practice guidelines (7). LOP is defined as the minimum pressure required for full arterial occlusion(7-9). PTP will be calculated at the start of every session. Prior to exercise, the cuff will be placed on the most proximal portion of the affected lower-limb and PTP calculated with the participant laying semi recumbent in a supine position (hip angle 120°). PTP will be determined using an automatic personalised tourniquet procedure within the system (Delfi Medical, Vancouver, BC, Canada). This procedure is designed to automatically calculate LOP with clinically acceptable accuracy and high reliability (9-11).

The system is comprised of a contour nylon cuff, connected by airtight hose tubing to a personalised tourniquet system (11, 12). During the PTP assessment, the device increases cuff pressure in step-by-step increments. To establish LOP the pneumatic pressure pulsations in the cuff bladder are analysed against the arterial pressure pulsations at each cuff pressure increment (12). The PTP will be 80% of the LOP value.

Using the tempo of training previously described (1-0-1), the total cuff inflation duration per exercise is 4 minutes (30reps + 30sec recovery + 15 reps + 30sec recovery +15reps +30sec recovery +15reps). The inflation pressure (80% LOP) will be maintained for the duration of the exercise and then deflated for 3 min to allow the patient to move to the next exercise/equipment station. It will then be re-inflated prior to commencing the second exercise (Knee extension). Thus, the total time under the inflated cuff will be 8 minutes per training session. Our previous protocol paper demonstrated that 8 minutes of BFR training performed twice daily during a busy 3-week admission was both feasible and 100% adhered to by the patient (4).

[1] The model demonstrating the prescribed resistance exercise in Figure 1 is a volunteer member of staff from the Defence Medical Rehabilitation Centre, Stanford Hall, UK. This volunteer provided full consent to include this image in the supplementary file.

Group 2: Heavy Load Resistance Training (HL-RT)

Participants will be asked to perform 4 sets of 12 repetitions of each exercise at ~70% of their predicted 1-RM with an inter-set interval of 2 minutes. This sequence of exercise will be repeated 3 times per week, amounting to 9 training sessions during the 3-week residential admission.

Determining Intervention Load

1-RM will be predicted using the 5-RM strength assessment routinely administered across Defence Rehabilitation and detailed as one of the outcome measures for the clinical-based RCT. The use of multiple repetitions (5-RM) lower-limb strength assessments have been shown to accurately predict 1-RM (13, 14). The loads lifted (Group 1: 70% 1-RM, Group 2: 20% 1-RM) will be determined using a prediction model commonly used by exercise practitioners (13).

Determining Load Progression and Regression

Assuming patients adapt over the three-week residential programme, the predicted 1RM is expected to increase and therefore we would expect to increase the weight lifted by small increments (e.g. 2.5 kg increases per week). Any increase in weight lifted will be at the discretion of the Exercise Rehabilitation Instructor (ERI), physiotherapist and participant, with training load (the number of repetitions and load lifted) for each session monitored and recorded accordingly. Clinician guidance for load progression/regression:

Regression: Exercises should be regressed if the participant (1) cannot complete 80% of total volume, (2) is unable to perform the exercise with correct technique or (3) the participant experiences an increase in injury symptoms either during or within 24 hours following an exercise session. In this case, the exercise load will be reduced. Should unilateral bodyweight movement (without external load) not be feasible, patients will be encouraged to use the other limb to assist. However, the objective will be to progress to unilateral loading as soon as the patient is capable.

Progression: Graded progression of load if participant completes sets/reps with more than 2 reps in reserve (RIR) on the final set and without aggravating symptoms. *Question: At the end of that final set, do you think you could you do another 2 reps? Yes/No*

Participant Monitoring Booklet

A participant monitoring booklet (Table 1) will enable daily monitoring of training load, participant wellness scores, symptomatic knee pain and localised muscle discomfort. This will be recorded in a participant booklet/diary that is completed daily by the patient and therapist.

Table 1: Participant Monitoring Booklet

Domain	Measure	Frequency
Participant Wellness Score	Likert scale (0-5) of 5 dimensions	Once daily, AM
Training load	Sets, reps, load completed	Immediately post study intervention session
Session rate of perceived exertion (sRPE)	Scale of 0 to 10	Immediately post study intervention session
Symptomatic knee pain	Visual analogue scale (VAS), 100 mm horizontal line	Immediately prior to starting the exercise, during the exercise and then 5 min post-exercise
Muscular discomfort	Visual analogue scale (VAS), 100 mm horizontal line	Immediately prior to starting the exercise, during the exercise and then 5 min post-exercise.
Symptomatic knee pain during a pain provoking functional task (single-leg knee bend)	Visual analogue scale (VAS), 100 mm horizontal line	Every third session for LL-BFR group. Every session for HRT session.

Muscular Discomfort and Symptomatic Knee Pain

A visual analogue scale (VAS) will be used to measure pain intensity. The VAS uses a 100 mm horizontal line anchored by the terms ‘no pain’ (0) and ‘worst possible pain’ (100). The VAS response format has shown good internal consistency, is easy to understand, is in wide clinical use, and has been sufficiently evaluated in clinical trial (15). Levels of muscular discomfort and symptomatic knee pain will be recorded immediately prior to starting the exercise, during the exercise and then 5 min post-exercise. Recent studies have demonstrated a hypoalgesia response up to 45 min post BFR training (16). Therefore, all participants will also be asked to score symptomatic knee pain during a pain provoking functional task (single-leg knee bend) immediately prior and 10 minutes following the cessation of exercise. These pain-related outcome measures will be repeated at the start, middle and end of each treatment week to monitor how pain response changes over time to both intervention groups. Muscular discomfort and symptomatic knee pain will be monitored during the 3-week rehabilitation admission using a participant monitoring booklet (table 1).

SECTION 2: REGIONAL REHABILITATION COURSE

The Regional Rehabilitation Unit (RRU) courses include the following key components which are further outlined within the document:

a. Assessment and outcome measure collection.
b. Education.
c. Group exercise therapy.
e. Individual exercise programming.
f. Adjuncts to rehabilitation

Timetabling and course capacity will be adapted to suit regional infrastructure constraints such as equipment, staffing, room availability and any social distancing restrictions. The residential rehabilitation course is a semi-structured programme that can be individualised to each patient according to physical assessment findings and response to treatment.

Both groups will attend all sessions within the residential rehabilitation course. The only component of treatment that differs between the two groups is the resistance training intervention prescribed to the quadriceps-based resistance exercises.

Assessment and Outcome Collection

Admission assessment. On course admission, in addition to the study outcome measures (outlined in separate documentation), the patient undergoes an assessment conducted by the clinical team (Physio and ERI) who are managing the patient's care whilst on course. The specific aims of the admission assessment are:

a. Identify key subjective/objective markers and biopsychosocial factors contributing to the patient's condition.
b. Establish patient's impressions, expectations and goals.
c. Assess functional movement patterning.
d. Record standardised measures of physical capacity through a combination of Patient Reported Outcome Measures (PROMs) and objective testing outlined within the ADAPT Study Outcome Measures protocol.
e. Establish any additional IDT needs and arrange appointments where required. This includes: MO, SEM Consultant, Clinical Specialist, Podiatry.

An individualised course plan should be devised and agreed with the patient. Admission assessment will be recorded within the patient's medical records.

On-course clinical reviews. To assess progress during the course, one to one patient reviews occur throughout the course (Physio and ERI) either formally or informally. These reviews aim to:

a. Ensure that the Individual Programme (IP) has been optimised and progressed / regressed as appropriate for each patient.
b. Monitor symptoms and key subjective / objective markers.
c. To identify early those patients who may require further intervention from the IDT.
d. To ensure that the patient has a clear understanding of their condition
e. To refine the patient goals, where required.

Discharge clinic. Prior to departing course all patients will undergo some form of discharge clinic with the Physio and ERI responsible for their care. This will include a course review, finalise IP and agree a rehabilitation plan for the following 3 months. This will be recorded on the patient's medical records.

Education

Patient education is the focal component of the course with the aim to equip patients with knowledge, tools and strategies to manage their rehabilitation. Thus, an educational component is incorporated into all lessons throughout the course.

The educational package within residential rehabilitation courses include several educational techniques to provide information in a format that suits a variety of learning styles. Delivery may include PowerPoint presentation, workshop format, one-to-one coaching and experiential group learning during group classes.
Specifically, the educational package includes:

a. **Opening brief.** To discuss the broad aims and objectives of the course and key safety aspects.
b. **Goal setting.** A presentation and/or practical session which stresses the importance of goals, compliance, motivation and self-efficacy. Patients set their own SMART goals which are reviewed as the course progresses.
c. **Anatomy of the Lower Limb.** Presentation which gives key functional anatomical and movement information to aid understanding of conditions and exercise approaches.
d. **Pain Management.** Presentation regarding the basic physiology and psychosocial aspects of pain and strategies to manage it.
e. **Principles of Fitness and Programme Design** Presentation and workshop covering training principle and programme design in relation to rehabilitation. Workshop to discuss and devise personal post course rehabilitation programme.
f. **Diet and nutrition.** Presentation aimed to educate personnel about healthy eating and the components of a balanced diet.

i. **Functional Strength training.** Practical session which covers the basic functional strength training principles, with a focus on derivatives of squat, hinge, single leg/split stance-based movement patterning.
j. **Cardiovascular.** Short presentation and then practical session providing basic information on heart rate training zones and CV training programmes.

Group Exercise Therapy Sessions

Group exercise therapy session provide a broad overview to introduce the patient safely to the different aspects of rehabilitation. Session format is a short education piece (verbal brief) and then practical session. The clinical staff ensure that suitable exercise progression and regression are catered for. The following are key components of the group exercise therapy sessions. It is understood these will be adapted to suit regional infrastructure and specific group needs.

a. Range of movement, flexibility and general mobility.
b. Sensorimotor training, balance and proprioception
c. Functional strengthening
d. Motor control and dynamic stability training
e. Cardiovascular

Individual Exercise Programme (IP) Sessions

The patient develops their IP throughout the course, which is reviewed and refined during individual programme sessions. Effective and personalised programming of their rehabilitation exercises is discussed during both group-based education sessions and on a one-to-one basis. On discharge the patient will take with them:

a. A finalised documented IP.
b. An IP log book that accommodates for their work/family/social commitments.

These are reviewed by clinical staff to ensure patient understanding.

Adjuncts to rehabilitation

Clinicians may utilise adjuncts to rehabilitation to reduce pain in the short term can include the following: manual therapy, taping, foot orthoses. Rehabilitation adjuncts are used as part of a combined intervention approach alongside the patients exercise programme and not used in isolation. This best represents typical management of persistent knee pain allowing clinicians to select treatment components that address specific impairment identified in individual patients. All adjuncts to rehabilitation to be documented within the participant monitoring booklet.

a. **Manual Therapy** Manual therapy interventions can be utilised as an adjunct to a patient's rehabilitation programme, however where possible patients should be taught self-management strategies. Manual therapy may include patellofemoral and knee mobilisations and manual soft tissue techniques (e.g. Ischaemic compression to peripatellar, retro patella or myofascial techniques). The addition or removal of techniques is at the discretion of the physiotherapist based upon re-assessment and patient progress.
b. **Taping** Clinicians may use tailored taping as an adjunct to a patient's rehabilitation programme to assist in pain reduction in the short term.
c. **Orthoses** Clinicians may prescribe prefabricated foot orthoses for patient with greater than normal pronation to reduce pain in the short term.

SECTION 3: REHABILITATION FOLLOWING COURSE

Individualized Exercise Programme (IP) Development

Upon course discharge, all participants will be instructed to perform unsupervised exercises outlined in an individualised exercise programme (IP) for a 12-week period prior to their final follow up which will be recorded in an IP log book.

To reflect "real-world" clinical practise, the participant's IP follows a semi-structured format. This is to ensure that the programme is standardized in order to be methodologically reproducible and reportable yet can be individualized dependant on the clinician's clinical findings and participant's rehabilitation goals (17, 18). This multimodal IP is based on best practise guidelines from within and outside of UK Defence rehabilitation, and current published evidence in this field. Specific guidance on the choices and constraints for selection and dosage of home exercises is provided.

Individualized Exercise Programme (IP) Parameters

Individualised exercise p*rogramme goals:*

1. Restore & improve knee muscle strength
2. Restore & improve hip/proximal muscle strength
3. Restore and improve lower body multi-joint movement and functional strength

Optional additional programme goals:

4. Restore & improve trunk/core strength and function[2]
5. Restore & improve ankle/distal strength[1]
6. Restore & improve sensorimotor and neuromuscular control [1]
7. Address any Range of Movement (ROM) deficits[1]

It is out of the scope of the intervention guidelines to advise on cardiovascular fitness and maintenance of non-injured areas. This is completed at the discretion of the treating clinician for the participant to meet British Armed Force Physical Employment Standards.

IP Development and Participant Education: During the residential rehabilitation course, the study-site clinician will discuss, develop and agree with the participant an individualised exercise programme (IP) to continue for the 12-weeks following course. Whilst on course the participant will be instructed on how to perform the exercises and supervised to ensure correct technique and implementation.

Environment: The IP may be completed at home or within a gym environment. The training location will be documented within the participant's IP log book.

[2] *To consider when physical deficits identified during initial clinical assessment. Capacity assessment based on Defence Rehabilitation best practice guideline recommendations for entry criteria to return to run. Refer to Annex 1.*

Supervision: After course discharge, the IP will be completed for 12-weeks unsupervised. Whilst on course, the participant will be coached and supervised by a member of the clinical team to ensure correct technique and implementation of the IP.

Frequency: Exercises will be performed a minimum of 2 and a maximum of 4 times a week. The IP includes 4 to 6 exercises per session meeting the following general requirements. When selecting the number of exercises per session the clinician will consider participant's compliance and time availability.

Exercise Selection: The exercises selected should aim to meet the programme goals 1-3 and be based upon the highest clinical priority established at the initial assessment. Exercises that meet programme goals 4-7, may be selected in addition if physical deficits are identified during the participants clinical assessment.

Dosage:

a. For resistance-based exercises and the early development of muscular strength, the recommended dosage will start at 3-4 sets of 10-12RM (~70% 1 RM) with 2-3 minutes rest between sets (weeks 0-4). To achieve graded load progression, the participant will be advised to increase load and decrease volume to 3-4 sets of 8-10 repetitions (~75-80% 1RM) for weeks 5-8, and 3-4 sets of 5-8 repetitions (~80-90% 1RM) for weeks 9-12. A 5-rep max test will determine initial and progressive load, this will be adjusted as per progression/regression parameter criteria below.
b. For stretching exercises, the recommended dose is 2-minutes total for each stretch, comprising 2 or more repetitions with 20-to-60 second hold times.
c. For functional sensorimotor and neuromuscular control drills the recommended dose is x 2 exercises for 4 'practices' x 30 seconds (4 minutes total).

Programme Regression: Exercises should be regressed if the participant (1) cannot complete 80% of total volume, (2) is unable to perform the exercise with correct technique or (3) the participant experiences an increase in injury symptoms either during or within 24 hours following an exercise session. In this case, the exercise load will be reduced.

Programme Progression:

a. For resistance-based exercises, graded progression of load will occur if participant completes sets/reps with more than 2 reps in reserve (RIR) on the final set and without aggravating symptoms. Question: At the end of that final set, do you think you could you do another 2 reps? Yes/No
b. For sensorimotor and neuromuscular control drills the most challenging drills / exercises that the patient can complete safely should be selected.

Programme modification: Recommended parameters may be adjusted to meet the participant's functional level and goals. Any modifications will be reported by the clinical team.

IP Adherence: The patient's adherence to the IP is discussed to identify individual barriers. Any obstacles to treatment implementation should be addressed by the clinician on course. Patient adherence will be recorded in an IP log book.

ANNEXES

ANNEX 1   Individualised Exercise Programme (IP) Session Planning and Selection Sheet

ANNEX 1 Individualised Exercise Programme (IP) Session Planning and Selection Sheet

**Treatment Goal - 1**
**Restore & improve knee muscle strength**

**Exercise**	**Tick to select**	**Dosage** (sets/reps/rest)	**Frequency** (sessions per week)	**Load** Bodyweight: double leg or single leg Theraband: colour Weight: Kg
Considerations: Quadriceps based exercise should not exacerbate symptoms (attention to open chain terminal knee extension in PFP participants). Knee exercises include both weight bearing and non-weight bearing exercises				
Quadriceps Strength				
Seated knee extension				
Inner range quadriceps				
Straight leg raise				
Single leg press				
Wall squat				
Hamstring Strength				
Seated knee flexion				
Hamstring bridge				
Romanian deadlift (RDL)				
Nordic curls				

**Treatment Goal - 2**
**Restore & improve hip/proximal muscle strength**

**Exercise**	**Tick to select**	**Dosage** (sets/reps/rest)	**Frequency** (sessions per week)	**Load** Bodyweight: double leg or single leg Theraband: colour Weight: Kg
**Considerations:** Hip exercises target hip extensor, external rotator and abductor muscle groups. Hip exercises include both weight bearing and non-weight bearing exercises				
Side lying hip abduction				
Side plank				
Banded side steps				
Glute bridge				
Hip thrust				
4-pt kneeling hip extension				
Standing hip extension				
Romanian deadlift (RDL)				
Arabesque				

**Treatment Goal – 3**

Restore and improve lower body multi-joint movement and functional strength

Exercise	Tick to select	Dosage (sets/reps/rest)	Frequency (sessions per week)	Load Bodyweight: double leg or single leg Theraband: colour Weight: Kg
**Considerations:** Participant instructed and able to demonstrate correct movement technique/patterning prior to addition of load.				
Back Squat				
Front Squat				
Lunge				
Deadlift				
Hex-bar deadlift				
Step ups				
Step Downs				
Lateral Step up				

Additional Treatment Goals

To consider when physical deficits identified during initial clinical assessment. Capacity assessment based on Defence Rehabilitation best practice guideline entry criteria to commence a return to run programme.

Additional Treatment Goal – 1
Restore & improve trunk/core strength and function

Exercise	Tick to select	Dosage (sets/reps/rest)	Frequency (sessions per week)	Load Bodyweight: double leg or single leg Theraband: colour Weight: Kg
**Considerations:** *Consider when deficits exist* *Capacity assessment: Side bridge test less than 60 secs to fatigue or less than 85% of non-symptomatic side.* Ensure adequate control of the pelvis and spine is maintained during core activity.				
Plank				
Side Plank				
Pallof Press				
Woodchop (high to low)				
Woodchop (low to high)				
Standing Rotation				
Farmers Carry				

**Additional Treatment Goal – 2**
**Restore & improve ankle/distal strength**

**Exercise**	**Tick to select**	**Dosage** (sets/reps/rest)	**Frequency** (sessions per week)	**Load** Bodyweight: double leg or single leg Theraband: colour Weight: Kg
**Considerations:** *Consider when deficits exist* *Capacity assessment: Calf and soleus raise test less than 20 reps or less than 85% of non-symptomatic side.*				
Standing heel raise (straight leg)				
Standing heel raise (bent leg)				
Seated heel raise (bent leg)				

**Additional Treatment Goal – 3**
**Restore & improve sensorimotor and neuromuscular control**

**Exercise**	**Tick to select**	**Dosage** (sets/reps/rest)	**Frequency** (sessions per week)	**Modification** Bodyweight: double leg or single leg Surface: Stable, unstable Description of movement perturbation
**Considerations:** *Consider when deficits exist.* *Capacity assessment: Single leg athletic stance (level 2) less than 30 secs or less than 85% of non-symptomatic side*				
Single leg balance				
Movement perturbation exercise				

**Additional Treatment Goal – 4**
**Address Range of Movement (ROM) Deficits.**

**Exercise**	**Tick to select**	**Dosage** (sets/reps/rest)	**Frequency** (sessions per week)	**Modification** Movement plane and description of stance
**Considerations:** *Consider when deficits exist.* *Capacity assessment: Muscle length test of passive range of motion less than 85% of non-symptomatic side.*				
Mobility target:				
Knee				
Hip				
Ankle				
Muscle Length target:				
Quadriceps				
Hamstrings				
Hip Flexors				
Glutes				
Gastrocnemius/Soleus				

References

1. Hoffmann TC, Glasziou PP, Boutron I, Milne R, Perera R, Moher D, et al. Better reporting of interventions: template for intervention description and replication (TIDieR) checklist and guide. BMJ : British Medical Journal. 2014;348:g1687.
2. Slade SC, Dionne CE, Underwood M, Buchbinder R. Consensus on Exercise Reporting Template (CERT): Explanation and Elaboration Statement. Br J Sports Med. 2016;50(23):1428-37.
3. Slysz J, Stultz J, Burr JF. The efficacy of blood flow restricted exercise: A systematic review & meta-analysis. Journal of science and medicine in sport. 2016;19(8):669-75.
4. Ladlow P, Coppack RJ, Dharm-Datta S, Conway D, Sellon E, Patterson SD, et al. Low-Load Resistance Training With Blood Flow Restriction Improves Clinical Outcomes in Musculoskeletal Rehabilitation: A Single-Blind Randomized Controlled Trial. Frontiers in physiology. 2018;9:1269-.
5. Ladlow P, Coppack RJ, Dharm-Datta S, Conway D, Sellon E, Patterson SD, et al. The effects of low-intensity blood flow restricted exercise compared with conventional resistance training on the clinical outcomes of active UK military personnel following a 3-week in-patient rehabilitation programme: protocol for a randomized controlled feasibility study. Pilot and Feasibility Studies. 2017;3(1):71.
6. Garber CE, Blissmer B, Deschenes MR, Franklin BA, Lamonte MJ, Lee IM, et al. American College of Sports Medicine position stand. Quantity and quality of exercise for developing and maintaining cardiorespiratory, musculoskeletal, and neuromotor fitness in apparently healthy adults: guidance for prescribing exercise. Medicine and science in sports and exercise. 2011;43(7):1334-59.
7. Patterson SD, Hughes L, Warmington S, Burr J, Scott BR, Owens J, et al. Blood Flow Restriction Exercise: Considerations of Methodology, Application, and Safety. Front Physiol. 2019;10:533.
8. AORN. Recommended practices for the use of the pneumatic tourniquet in the perioperative practice setting. Aorn j. 2007;86(4):640-55.
9. Loenneke JP, Allen KM, Mouser JG, Thiebaud RS, Kim D, Abe T, et al. Blood flow restriction in the upper and lower limbs is predicted by limb circumference and systolic blood pressure. Eur J Appl Physiol. 2015;115(2):397-405.
10. McEwen JA, Owens JG, Jeyasurya J. Why is it Crucial to Use Personalized Occlusion Pressures in Blood Flow Restriction (BFR) Rehabilitation? Journal of Medical and Biological Engineering. 2019;39(2):173-7.
11. Loenneke JP, Fahs CA, Rossow LM, Sherk VD, Thiebaud RS, Abe T, et al. Effects of cuff width on arterial occlusion: implications for blood flow restricted exercise. Eur J Appl Physiol. 2012;112(8):2903-12.
12. Hughes L, Rosenblatt B, Gissane C, Paton B, Patterson SD. Interface pressure, perceptual, and mean arterial pressure responses to different blood flow restriction systems. Scand J Med Sci Sports. 2018;28(7):1757-65.
13. Abadie BR, Wentworth MC. Prediction of one repetition maximal strength from a 5-10 repetition submaximal strength test in college-aged females. Journal of exercise physiology online. 2000;3(3):1-8.
14. Dohoney P, Chromiak JA, Lemire D, Abadie BR, Kovacs C. Prediction of one repetition maximum (1-RM) strength from a 4-6 RM and a7-10 RM submaximal strength test in healthy young adult males. Journal of exercise physiology online. 2002;5(3):54-9.
15. Collins SL, Moore RA, McQuay HJ. The visual analogue pain intensity scale: what is moderate pain in millimetres? Pain (Amsterdam). 1997;72(1):95-7.

16. Korakakis V, Whiteley R, Giakas G. Low load resistance training with blood flow restriction decreases anterior knee pain more than resistance training alone. A pilot randomised controlled trial. Physical therapy in sport. 2018;34:121-8.
17. Skivington K, Matthews L, Simpson SA, Craig P, Baird J, Blazeby JM, et al. A new framework for developing and evaluating complex interventions: update of Medical Research Council guidance. BMJ. 2021;374:n2061.
18. Bennell KL, Hinman RS. A review of the clinical evidence for exercise in osteoarthritis of the hip and knee. J Sci Med Sport. 2011;14(1):4-9.
