# Supplementary material for: ADAPTations to low load blood flow restriction exercise versus conventional heavier load resistance exercise in UK military personnel with persistent knee pain: protocol for the ADAPT study, a multi-centre randomized controlled trial
Source: BMC Musculoskelet Disord. 2023 Jul 17;24:580. doi: 10.1186/s12891-023-06693-3 (PMC10351180; doi:10.1186/s12891-023-06693-3)
Supplement: Supplementary file 2 — Additional file 2. ADAPT Study Outcome Measures. [file 12891_2023_6693_MOESM2_ESM.pdf]

ADAPT Study Outcome Measures

Main Randomised Controlled Trial Outcome Measures

Patient Reported Outcome Measures / Questionnaires

Ser	Outcome Measure / Instrument	Measurement Timepoint	Administrator	Comments / Construct
1	Demographics Questionnaire	T1	REDCap Survey Queue	Demographic Characteristics
2	Health Anxiety Depression Scale (HADS)	T1	REDCap Survey Queue	Psychological Health
3	Pain Medication Record Sheet	T1, T2, T3	REDCap Survey Queue	Pain Medication Record
4	Lower Extremity Functional Scale (LEFS)	T1,T2,T3	REDCap Survey Queue	Knee Specific Function
5	International Physical Activity Questionnaire (IPAQ)	T1, T3	REDCap Survey Queue	Physical Activity Levels
6	Musculoskeletal Health Questionnaire (MSK-HQ)	T1,T2,T3	REDCap Survey Queue	Overall MSK Health
7	Knee Injury and Osteoarthritis Outcome Score (KOOS)	T1,T2,T3	REDCap Survey Queue	Knee Specific Function
8	Patient Specific Functional Scale	T1,T2,T3	REDCap Survey Queue	Patient specific functional deficits
9	Tampa Scale of Kinesiophobia (TSK)	T1,T2,T3	REDCap Survey Queue	Kinesiophobia
10	Sports Injury Rehabilitation Beliefs Survey (SIRBS)	T1,T2,T3	REDCap Survey Queue	Self-Efficacy
11	Numeric Pain Rating Scale (NPRS)	T1,T2,T3	REDCap Survey Queue	Generic Pain
12	Functional Activity Assessment (FAA)	T1,T2,T3	REDCap Survey Queue	Occupational Function

Physical / Functional Capacity Tests

13	Decline Knee Bend	T1,T2, T3	PT / ERI	Non-symptomatic loaded knee flexion
14	5-RM Lower-Limb Strength: Single Leg Press	T1,T2, T3	PT / ERI	Lower-limb muscle strength
15	Isometric Muscle Strength: Hip Extension, Hip Flexion, Hip Abduction, Hip Adduction, Knee Extension, Knee Flexion	T1,T2, T3	PT / ERI	Hip and knee isometric muscle strength in single movement planes
16	Single Leg Heel Raises to Fatigue	T1, T2, T3	PT / ERI	Muscular endurance
17	Qualitative Assessment of Single Leg Squat (QASLS)	T1,T2, T3	PT / ERI	Lower-limb postural control/stability

Nested Mechanistic Outcome Measures

17	Imaging:<br>• MRI: cross-sectional area and volume of the quadriceps femoris and hamstrings<br>• Ultrasound: Vastus lateralis pennation angle and fascicle length<br>• Physiological cross-sectional area of the vastus lateralis	T1,T2, T3	RS	Muscle morphology and architecture
18	Neuromuscular Performance:<br>• Isometric mid-thigh pull (with sEMG)<br>• Maximum Isometric Voluntary Contraction (with sEMG)<br>• Countermovement Jump (with sEMG and 3D motion capture)<br>• 60 º Bilateral Squat (with sEMG and 3D motion capture)<br>• 60 º Unilateral Squat (with sEMG and 3D motion capture)	T1,T2, T3	RS	Neuromuscular performance
19	Physiological responses:<br>• Venepuncture	T1,T2, T3	RS / N	Muscle damage, inflammation, oxidative stress and vascular health

† T1=Course Admission;T2= Course Discharge;T3=3-month follow-up
* PT = Study Physiotherapist; ERI=Exercise Rehabilitation Instructor, RS = Research Staff, N = Nurse

Patient Reported Outcome Measures / Questionnaires

All clinical outcome measures will be assessed at course admission, course discharge and at 3 months following course. The functional outcome measurements detailed below are collected as part of routine clinical practice and will be conducted and recorded by an experienced supervising therapist.

Participant Demographics Questionnaire

Baseline characteristics will be collected upon admission and will include; participant entry questionnaire and Health Anxiety Depression Scale (HADS). Personal and demographic characteristics including age, body height, body mass, body mass index (BMI), duration of symptoms, previous injuries, previous treatment, level of psychological impairments (via HADS questionnaire), military occupation, duration of military service, smoking and drinking habits will be obtained via the ADAPT Study Demographics Questionnaire, distributed and stored using REDCap.

Pain Medication Questionnaire

Patient will be asked to report their current pain medication at course admission, course discharge and at 3-momnths following course.

Patient Reported Outcome Measure (PROM) Questionnaires

The subjective PROMs taken at T1, T2 and T3 will include; Lower Extremity Functional Scale (LEFS), Knee Injury and Osteoarthritis Outcome Score (KOOS), Patient Specific Functional Scale, Musculoskeletal Health Questionnaire (MSK-HQ), Tampa Scale of Kinesiophobia (TSK), Numeric Pain Rating Scale (NPRS), Functional Activity Assessment (FAA), Sports Injury Rehabilitation Beliefs Survey (SIRBS) and Physical Activity Questionnaire (T1 and T3 only). All questionnaires will be distributed via email using REDCap.

Physical / Functional Capacity Tests

The following physical/functional capacity tests are presented in the order of testing.

Warm Up: Progressive warm-up on a stationary bike for 5 minutes. If bike aggravates the participant's symptoms an alternative CV equipment will be identified by the assessing clinician.

Maximum Knee Flexion Angle During Decline Knee Bend Task

The therapist will palpate the greater trochanter and the patient will be instructed to keep the fingertips of the ipsilateral hand on the greater trochanter. The participant will be allowed to use their other fingertip (contralateral hand) against the wall to prevent postural and movement control issues or fear. Participant will stand in unilateral stance (shoes off) on the decline board (25º) and be instructed to descend into knee flexion by keeping the knee above the foot. They will be asked to pause for once second at the end of their range and then stand upright adopting the start position (see figure 1).

This will be repeated three times. The maximum knee flexion angle will be determined using video analysis. The therapist will record a video of the participant performing the test using the study tablet device (Samsung Galaxy Tab A7 Lite) with the camera positioned to the side of their body (sagittal plane) perpendicular to the knee at 1m distance. The image being analysed will be framed to ensure no facial features are identifiable. The image will include a side profile of the participant's feet up to their torso. The video file will be uploaded directly onto REDCap. This video will then be analysed by ADMR research staff using DartFish technology. The landmarks used to identify knee angle include the lower point of the lateral malleolus, half the distance between patella and the hollowest point of the knee cavity and the middle finger of the participant marking the centre of the greater trochanter.

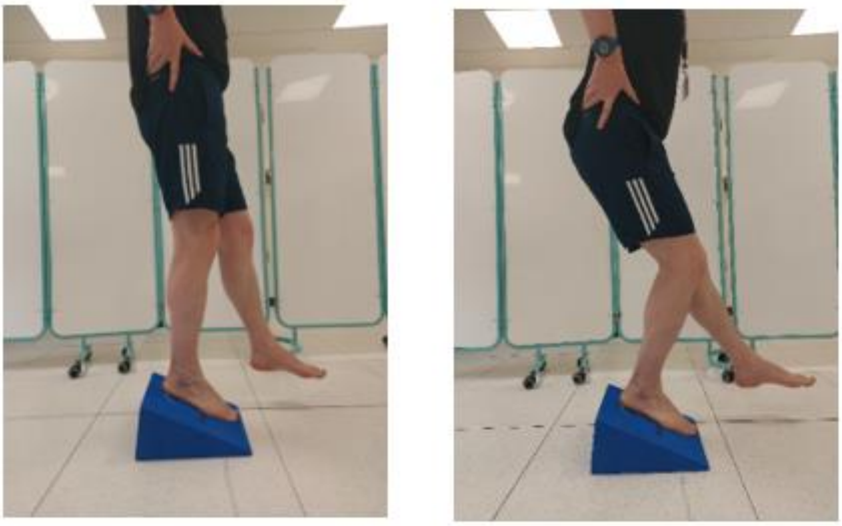

A. **B.**

Figure 1: Decline knee bend task. A, Start position; B, End position

Single Leg Press (5RM)

Unilateral muscle strength will be assessed using a dynamic 5-RM test performed on the leg press. An initial load is set by the assessing clinician based on the result of a clinical assessment, pain intensity and participant feedback. Testing position is standardised so that at the bottom position, the participant's knee and hip are flexed to 90 degrees. Individual foot and seat position will be recorded to replicate posture for re-testing. The assessing clinician from each study centre will ensure that proper exercise technique and full range of motion (ROM) is achieved.

Participants will perform a double leg 10-repetition specific warm-up. This will be followed by successive attempts of single leg 5-RM testing at progressive levels of maximum effort, 1st set at 75%; 2nd set at 90% and subsequent sets at 100% effort until maximum load achieved for 5 complete reps. A 30 second to1 minute rest interval is required during the warmup and progressive effort lifts (75-90% effort); all testing at 100% maximum effort for 5RM will require a 2-minute rest interval between sets

* The authors acknowledge that due to the likely interference effect of a pain response, performing a 'maximum effort' physical task in a lower-limb injured cohort is unlikely to yield a true measure of MSK performance. It is more accurate to describe outcome scores as providing an 'indication' of participant performance and progression. We will highlight this as a potential weakness in our study but feel this is a challenge in the measurement of muscle force / strength in all MSK injury research. All outcomes measured will be implemented and recorded based upon a best effort at the time of assessment.

Isometric Hip and Knee Strength (HHD)

Measurements will be taken using a wireless digital Lafayette hand-held dynamometer (HHD) (Lafayette, Indiana, United States). The examiner will apply resistance in a fixed position whilst the participant exerts a 3 second isometric maximal voluntary contraction (MVC) against the dynamometer and the examiner.

i) Familiarisation

For familiarisation with the testing procedure, the participant is asked to perform one isometric sub-maximal contraction into the hand-held dynamometer, guided by the investigators' hand, to ensure that the correct action was performed. Then, an additional practice trial, in the form of an MVC against the HHD is completed.

ii) Maximum Testing

Participants will perform three consecutive attempts with a 30 second recovery between attempts. Strength measures will be reported as Newtons (N). The highest value of the 3 valid MVCs is used in the subsequent analyses. If the last MVC elicited the highest value, another MVC was performed until no further increase was measured. The verbal encouragement in the form of "go ahead-push-push–push-push and relax" is used during the contractions.

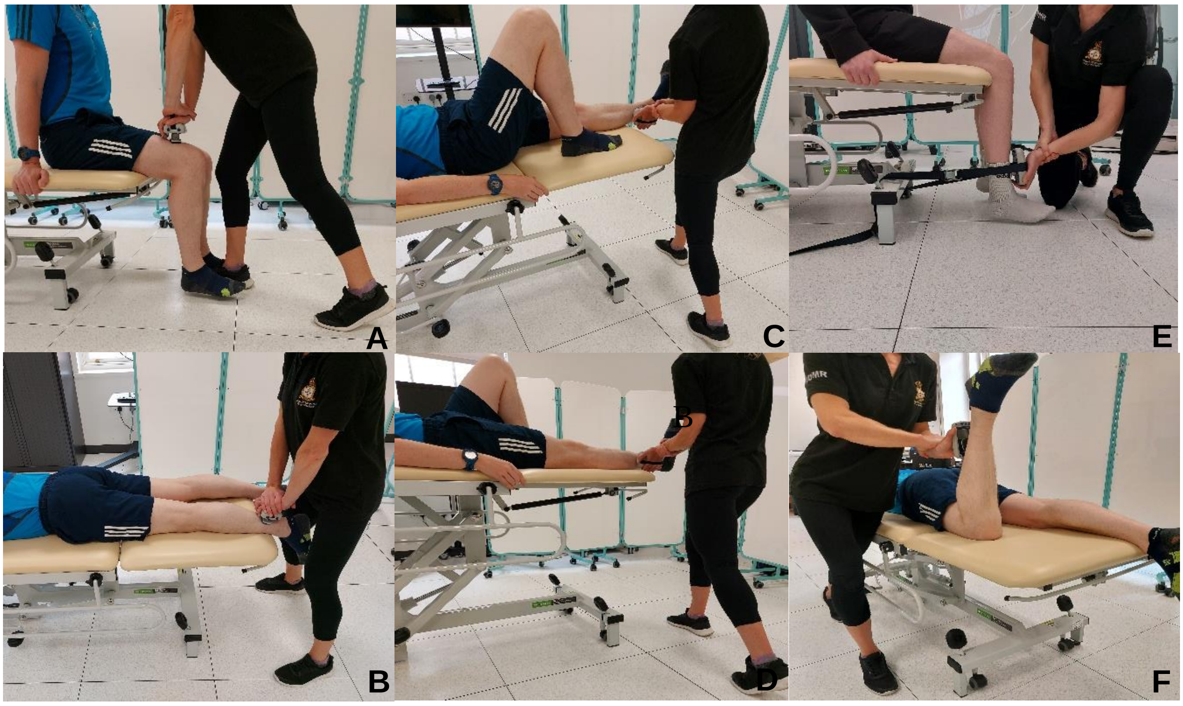

Figure 2: Testing positions and order for isometric strength assessment.

Legend:

A) Hip flexors with the participant seated and hips and knees flexed at 90°. The testing foot will be held just off the floor (~3cm). Dynamometer placed on the anterior aspect of the thigh, 5 cm proximal to the knee joint.

(B) Hip extensors with the participant lying prone and hips and knees extended. Dynamometer placed on the posterior aspect of the shank, 5 cm proximal to the ankle joint. The participant is instructed to extend at the hip ensuring the knee does not flex.

(C) Hip adductors with the participant lying supine with hip and knee extended in the testing limb and non-testing limb flexed so foot is placed on the plinth. Dynamometer placed on the medial aspect of the shank, 5 cm proximal to the ankle joint

(D) Hip abductors with the participant lying supine with hip and knee extended in the testing limb and non-testing limb flexed so foot is placed on the plinth hips and knees extended. Dynamometer placed on the lateral aspect of the shank, 5cm proximal to the ankle joint.

(E) Knee extensors with the participant seated and hips and knees flexed at 90°. The testing foot will be held just off the floor (~3cm). Dynamometer placed on the anterior aspect of the shank; 5 cm proximal to the ankle joint. **A belt will be used to stabilise the position; this will be secured to the plinth and attached to the dynamometer**

(F) Knee flexors with the participant lying prone, hips extended, and testing knee flexed at 90°. Dynamometer placed on the posterior aspect of the shank; 5 cm proximal to the ankle joint.

Single Leg Calf Raises to Fatigue

Participants take a single leg stance (shoes off) and then ankle plantar flex to raise their heel through full range of motion. The participant will be allowed to use their other fingertip (contralateral hand) against the wall to prevent postural and movement control issues or fear. Heel raises are performed to a metronome set at 60bpm tempo. The test concludes when:

1. The subject is unable to move through full range
2. The subject slows below the movement cadence (60bpm)

The patient is allowed one error where the clinician can prompt the patient with the following phrases "*raise your heel as high as possible*" or "*keep to the timing of the metronome*". The test concludes if the patient cannot keep to the movement standards. The test is completed

on the affected limb followed by the non-affected limb, with a 30s rest between tests. The test is quantified by the total number of raises performed.

Qualitative Assessment of Single Leg Squat (QASLS)

The participants will be asked to take a single leg stance (shoes off), arms by side, with raised leg held behind the body. The participant will then squat downwards at least 45° knee flexion and no greater than 60°, over a period of five seconds. Knee flexion angle will be checked during practice trials using a standard goniometer. Trials will only be accepted if the participant squatted within the desired range of knee flexion. Participant will be allowed a maximum of three practice trials prior to data collection. Scoring is defined by a zero for appropriate strategy and one for inappropriate movements, for each region with best overall score identified as a 0 and worst 10 points. The patient is scored between 0-10, with a higher score indicating a higher risk of injury or a poorer performance.

The therapist will record a video of the participant performing the test using the study tablet device (Samsung Galaxy Tab A7 Lite) with the camera positioned to the front of their body (sagittal plane) perpendicular to the knee at 1m distance. The image being analysed will be framed to ensure no facial features are identifiable. The image will include a front profile of the participant’s feet up to their torso. The video file will be uploaded directly onto REDCap. Two ADMR research staff involved in the study will independently assess the video of each subject performing the QASLS. Each video will be viewed three times at standard speed and then scored using the qualitative scoring sheet. Discrepancies in scoring will be reviewed by a third independent reviewer until a level of agreement is achieved.

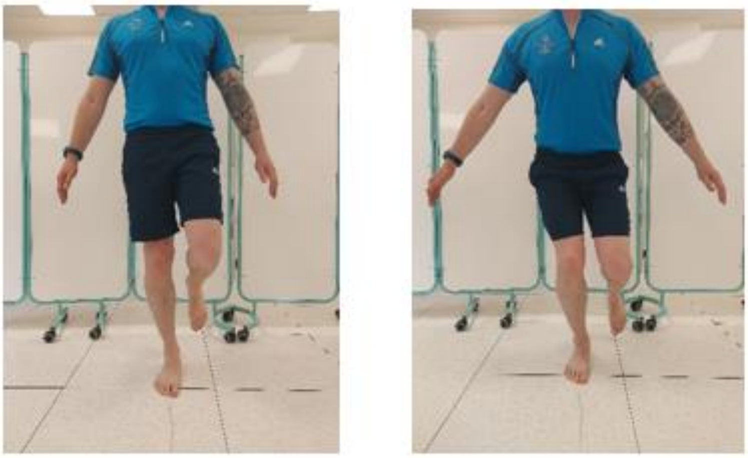

A. **B.**

Figure 3: QASLS. A, Start position; B, End position

Participant Monitoring Booklet

A participant monitoring tool (Table 1) will enable daily monitoring of training load, participant wellness scores, symptomatic knee pain and localised muscle discomfort. This will be recorded in a participant booklet/diary that is completed daily by the patient and therapist.

Table 1: Participant Monitoring Booklet

Domain	Measure	Frequency
Participant Wellness Score	Likert scale (0-5) of 5 dimensions	Once daily, AM
Training load	Sets, reps, load completed	Immediately post study intervention session
Session rate of perceived exertion (sRPE)	scale of 0 to 10	Immediately post study intervention session
Symptomatic knee pain	Visual analogue scale (VAS), 100 mm horizontal line	Immediately prior to starting the exercise, during the exercise and then 5 min post-exercise
Muscular discomfort	Visual analogue scale (VAS), 100 mm horizontal line	Immediately prior to starting the exercise, during the exercise and then 5 min post-exercise.
Symptomatic knee pain during a pain provoking functional task (single-leg knee bend)	Visual analogue scale (VAS), 100 mm horizontal line	Every third session for LL-BFR group<br>Every session for HRT session.

Muscular Discomfort and Symptomatic Knee Pain

A visual analogue scale (VAS) will be used to measure pain intensity. The VAS uses a 100 mm horizontal line anchored by the terms ‘no pain’ (0) and ‘worst possible pain’ (100). Levels of muscular discomfort and symptomatic knee pain will be recorded immediately prior to starting the exercise, during the exercise and then 5 min post-exercise. All participants will also be asked to score symptomatic knee pain during a pain provoking functional task (single-leg knee bend) immediately prior and 10 minutes following the cessation of exercise. These pain-related outcome measures will be repeated at the start, middle and end of each treatment week to monitor how pain response changes over time to both intervention groups. Muscular discomfort and symptomatic knee pain will be monitored during the 3-week rehabilitation admission using a participant monitoring tool.

Nested Mechanistic Study Outcome Measures

The following outcome measure assessments are presented in the order of testing.

Blood Sampling

A trained phlebotomist will draw blood from an antecubital vein into 2 x 10 ml vacutainer tubes (BD Biosciences, New Jersey, USA); one containing silica particles followed by one containing dipotassium ethylenediaminetetraacetic acid (K_2-EDTA). Tubes will be inverted upon sampling (5 – 6 and 8 – 10 inversions for silica particles and K_2-EDTA tubes respectively), placed upright for 60 min before centrifugation at 3,500 revolutions per minute for 10 min. Serum and plasma will then be separated, aliquoted and stored at -80 °C until further analysis.

Quadriceps Muscle Architecture

Participants will be assessed in a standardised testing position; they will lie supine with a towel placed underneath their knee to allow for a 40 º knee angle (measured using a goniometer) and will be instructed to relax to ensure the muscle is imaged at rest. Three measurements will be taken at 50 % of the superficial medio-lateral width of the VL at 35, 50 and 65 % of the distance between the lateral condyle of the femur and the greater trochanter. An extended field of view mode will be used to capture a 10 cm long image (5 cm proximal and distal to each ultrasound site), creating a panoramic view of the entire fascicle to ensure that the fascicular path does not extend beyond the acquired image. This mode of ultrasound is thought to be well suited to musculoskeletal imaging; it has displayed low measurement error and high inter- and intra-rater reliability during measures of VL architecture (1, 2). The transducer will be coated with a water-soluble transmission gel, with minimal pressure applied to the dermal surface to aid acoustic contact and minimise muscle deformation. The transducer will be orientated parallel to the muscle fascicles and perpendicular to the skin. This measurement technique is known to reduce error in architectural measurements (3-5).

To ensure measurement sites are identical between time points, imaging locations will be replicated based on the probes distance from external anatomical landmarks (greater trochanter, lateral condyle of the femur and the medial and lateral borders of the VL). The angle of the probe relative to the longitudinal axis of the femur will also be recorded and replicated. During image acquisition at T2 and T3, ultrasonographic images will be taken whilst referring to images captured at T1, with internal landmarks such as fat and connective tissue used to verify that probe location has been accurately reproduced.

Neuromuscular Performance

Warm Up: Progressive warm-up on a stationary bike for 5 min at a self-selected intensity.

Bilateral and Unilateral squat

For each type of squat, participants will be given 3 warm-up trials, where a goniometer will be aligned with the knee joint centre in the sagittal plane at 60 °. After 2 min rest, five trials will be performed, where participants will be asked to squat to a depth beyond 60 ° and return to an upright position over a 4 s metronome paced cycle. During the bilateral squat, participants will be instructed to hold their hands on their hips, with their feet positioned on separate force plates. During the unilateral squat, participants will be instructed to hold their non-stance limb at approximately 90 ° of knee flexion and have their hands by their side. If participants lose balance during the trial, causing part of the ipsilateral foot to lose contact with the force plate or causing the contralateral foot to make contact with the ground or force plate, the trial will be deemed invalid and repeated.

Countermovement jump

As part of a familiarisation process, five submaximal CMJ's will be performed at 50 % (x 2), 75 % (x 2) and 90 % (x 1) of perceived maximal exertion. Three maximal CMJ trials will then be performed. All familiarisation and maximal CMJ trials will be interspaced by 1 min recovery. Participants will be instructed to stand as still as possible in an upright position, hands on hips and with their feet positioned on separate force plates. On the command 'jump,' participants will rapidly squat to a self-selected depth followed by a maximal effort vertical jump. Upon landing, participants will squat to a self-selected depth before returning to the start position to signify the end of the test. Trials will be deemed invalid and will be repeated if participants remove their hands from their hips, show excessive knee flexion once airborne or land with part of their foot off the force plate.

Isometric mid-thigh pull

The IMTP will be performed using a previously established, standardised testing procedure (6). Prior to testing, the bar height will be adjusted to allow the participant to obtain optimal hip (140 – 150 º) and knee (125 – 150 º) angles, with 5 – 10 º of forward trunk inclination. These joint angles will be verified using a goniometer. Participants will be asked to stand on the force plates with their mid-foot under the bar and feet approximately hip width apart. Their knees will be in front of the vertical plane of the bar (creating ankle dorsiflexion) and thighs in contact with the bar. They will be asked to stand with an upright torso, with their

shoulders above or slightly behind the vertical plane of the bar and with their shoulders retracted and depressed. Bar height, foot positioning, grip width, trunk inclination, hip angle and knee angle will be recorded during testing at T1 and replicated at T2 and T3.

To become re-familiarised with the IMTP testing procedure, three submaximal IMTP trials will be performed which will consist of 3 s trials at 50, 75 and 90 % of perceived maximal effort, each interspaced by 1 min recovery. Participants will rest for 2 min before completing three maximum effort trials of 5 s duration with 2 min rest between attempts. Standardised instructions will be provided prior to each trial. Participants will be instructed to 'drive their feet into the ground as hard and as fast as possible'. A period of quiet standing will be performed in order to obtain a stable force baseline. During this period, participants will be instructed to 'apply just enough pre-tension to the bar to remove 'slack' from the body, without pulling the bar up into the mid-thigh pull rig'. Participants will be instructed to 'stand still' before being given the countdown '3, 2, 1 Drive'. Standardised verbal encouragement will then be provided throughout the duration of each trial. Lifting straps will be used to ensure grip strength does not limit performance. Each maximum effort trial will be visually inspected against the following criteria: the force during the quiet standing phase should be ≤ 50 N above body mass and not lower than body mass, peak force should not occur at the end of the trial and there should be no countermovement at the start of the trial (6). Additionally, participants will be observed during each trial to ensure that joint angles do not change. Any trials which violate these criteria will be repeated. Lastly, if peak force during the final trial is ≥ 250 N above the penultimate trial, additional trials will be performed until this threshold is not reached.

Maximum Voluntary Isometric Contraction

Participants will be seated in an isokinetic dynamometer with a hip angle of 90 °, and knee angles of 60 ° and 45 ° for knee extension and flexion respectively, as measured by a goniometer. The lateral condyle of the femur will be aligned with the axis of rotation of the dynamometer. Adjustable straps will be tightly fastened across the pelvis, shoulders and thigh to prevent extraneous movement. An ankle strap will be placed at ~ 15 % of the distance between the lateral malleolus to the femoral condyle. Seat configuration and dynamometer lever length will be recorded during testing at T1 and replicated at T2 and T3.

At the beginning of the neuromuscular testing, three familiarisation trials will be performed, consisting of 3 s trials at 50, 75 and 90 % of perceived maximal effort, each interspaced by 30 s recovery. Maximum voluntary isometric contraction data will then be collected as the final neuromuscular test and will begin with a warm-up, which will replicate the familiarisation

trials. Participants will then perform three, 3 s MVIC's of the knee extensors and knee flexors, with each trial interspaced by 2 min recovery. Standardised verbal instructions will be given during each trial; participants will be given the countdown '3, 2, 1' before being repeatedly encouraged to "push" (knee extension) or "pull" (knee flexion) as hard and as fast as possible. The highest instantaneous torque recorded during any MVIC will be defined as the knee extension or flexion maximum voluntary torque.

References

1. Franchi MV, Raiteri BJ, Longo S, Sinha S, Narici MV, Csapo R. Muscle Architecture Assessment: Strengths, Shortcomings and New Frontiers of in Vivo Imaging Techniques. Ultrasound in Medicine & Biology. 2018;44(12):2492-504.
2. El-Ansary D, Marshall CJ, Farragher J, Annoni R, Schwank A, McFarlane J, et al. Architectural anatomy of the quadriceps and the relationship with muscle strength: An observational study utilising real-time ultrasound in healthy adults. Journal of Anatomy. 2021;239(4):847-55.
3. Klimstra M, Dowling J, Durkin JL, MacDonald M. The effect of ultrasound probe orientation on muscle architecture measurement. Journal of Electromyography and Kinesiology. 2007;17(4):504-14.
4. Bénard MR, Becher JG, Harlaar J, Huijing PA, Jaspers RT. Anatomical information is needed in ultrasound imaging of muscle to avoid potentially substantial errors in measurement of muscle geometry. Muscle & Nerve. 2009;39(5):652-65.
5. Kwah LK, Pinto RZ, Diong J, Herbert R. Reliability and validity of ultrasound measurements of muscle fascicle length and pennation in humans: A systematic review. Journal of applied physiology (Bethesda, Md : 1985). 2013;114.
6. Comfort P, Dos'Santos T, Beckham GK, Stone MH, Guppy SN, Haff GG. Standardization and Methodological Considerations for the Isometric Midthigh Pull. Strength & Conditioning Journal. 2019;41(2):57.
