# Supplementary material for: ADAPTations to low load blood flow restriction exercise versus conventional heavier load resistance exercise in UK military personnel with persistent knee pain: protocol for the ADAPT study, a multi-centre randomized controlled trial
Source: BMC Musculoskelet Disord. 2023 Jul 17;24:580. doi: 10.1186/s12891-023-06693-3 (PMC10351180; doi:10.1186/s12891-023-06693-3)
Supplement: Supplementary file 3 — Additional file 3. ADAPT Study Eligibility Criteria. [file 12891_2023_6693_MOESM3_ESM.pdf]

INITIAL STUDY MIAC **INCLUSION CRITERIA**

**The following criteria MUST be answered YES for participant to be included in the trial (except where NA is appropriate):**		**Yes**	**No**	**N/A**
1.	Mechanical knee pain for at least three-months	☐	☐	☐
2.	Clinical signs and symptoms of knee pain arising from the tibiofemoral or patellofemoral joint (* refer to clinical diagnostic guide)	☐	☐	☐
3.	Reduced occupational employability secondary to knee pain	☐	☐	☐
4.	Progression of resistance training load within the patient's rehabilitation programme limited by knee pain.	☐	☐	☐
5.	Age ≥ 18-55yrs	☐	☐	☐
6.	Available to attend for the entire duration of the RRU course and a review appointment 3-months following course	☐	☐	☐
**If any of the above criteria is answered NO, the participant is NOT eligible for the trial and must not be included in the study.**				

INITIAL STUDY MIAC **CLINICAL DIAGNOSTIC GUIDE**

****Tibiofemoral Pain**	****Patellofemoral Pain**
Clinical and/or radiographic findings indicative of pain arising from the tibiofemoral joint, for example degenerative meniscal tears or early osteoarthritis within the tibiofemoral joint.<br><br>Diagnosis through exclusion of:<br>o Fracture/dislocation<br>o Instability from ligament insufficiency or rupture<br>o Tendinopathy<br>o Soft tissue injury<br>o non-local pain source<br>o Non-musculoskeletal or serious pathological condition	Pain around or behind the patella, which is aggravated by at least one activity that loads the patellofemoral joint during weight bearing on a flexed knee (e.g. squatting, stair ambulation, jogging/running, hopping/jumping)<br><br>Additional criteria:<br>o Crepitus or grinding sensation emanating from the patellofemoral joint during knee flexion movements<br>o Tenderness on patellar facet palpation<br>o Small Effusion<br>o Pain on sitting, rising from sitting, or straightening knee following sitting.<br>*(Crossley et al., 2016)

INITIAL STUDY MIAC **EXCLUSION CRITERIA**

**The following criteria MUST be answered NO for participant to be included in the trial:**		**Yes**	**No**
1.	Any medical contraindication related to BFR*	☐	☐
2.	Tibial, femoral or patella fracture +/- dislocation	☐	☐
3.	Instability resulting from ligament deficiency	☐	☐
4.	Patella dislocation (within the last 3-months) or recurrent patellar dislocation.	☐	☐
5.	Clinical signs and symptoms of patellar tendinopathy	☐	☐
6.	Planned surgery over the study period (3-month period).	☐	☐
7.	Cortico-steroid or intraarticular injection intervention into the knee within the previous 7-days	☐	☐
8.	Restricted range of movement (i.e., Chronically locked knee or fixed flexion deformity)	☐	☐
9.	Non-musculoskeletal or serious pathological condition (i.e., Inflammatory arthropathy, infection or tumour)	☐	☐
10.	Spinal or referred pain from non-local pain source	☐	☐
11.	Any physical impairment or co-morbidities precluding the safe participation in the rehabilitation programme and/or assessment procedures	☐	☐
12.	Previous knee surgery within the last 12 months to the affected limb	☐	☐
13.	Insufficient capacity to provide informed consent	☐	☐
**If any of the above criteria is answered YES, the participant is NOT eligible for the trial and must not be included in the study.**			

INITIAL STUDY MIAC **MEDICAL CONTRAINDICATIONS TO BFR**

**The following criteria MUST be answered NO for participant to be included in the trial:**		**Yes**	**No**
1.	History of cardiovascular disease including hypertension, peripheral vascular disease, thrombosis/embolism, ischaemic heart disease, myocardial infarction.	☐	☐
2.	History of the following musculoskeletal disorders: rheumatoid arthritis, avascular necrosis or osteonecrosis, severe osteoarthritis.	☐	☐
3.	History of the following neurological disorders: Peripheral neuropathy, Alzheimer’s disease, amyotrophic lateral sclerosis, Parkinson’s disease, severe traumatic brain injury.	☐	☐
4.	Varicose veins in the lower-limb.	☐	☐
5.	Acute viral or bacterial upper or lower respiratory infection at screening.	☐	☐
6.	Known or suspected lower limb chronic exertional compartment syndrome (CECS)	☐	☐
7.	Postsurgical swelling.	☐	☐
8.	Surgical insertion of metal components at the position of cuff inflation.	☐	☐
9.	History of any of the following conditions or disorders not previously listed: diabetes, active cancer.	☐	☐
10.	History of elevated risk of unexplained fainting or dizzy spells during physical activity/exercise that causes loss of balance	☐	☐
11.	History of haemorrhagic stroke or exercise induced rhabdomyolysis.	☐	☐
**If any of the above criteria is answered YES, the participant is NOT eligible for the trial and must not be included in the study.**			

VISIT 1 – INITIAL STUDY MIAC **PARTICIPANT ELIGIBILITY REVIEW**

**End of Screening Visit Checklist:**			
		**Yes**	**No**
**1.**	**Does the patient satisfy the inclusion and exclusion criteria?**	☐	☐
**2.**	**Has the participant received the study brief and patient information leaflets?**	☐	☐
**3.**	**Is the participant still willing to be contacted by the ADAPT research team to discuss study enrolment?**	☐	☐
**4.**	**Have the findings of the medical examination and study screening been documented in the patient's medical records?**	☐	☐
**5.**	**Are the patient's contact details correct within the patient's medical records?**	☐	☐

**Participant's eligibility SEM physician sign-off:**<br>**SEM physician's Name:** ___________________<br>**SEM physician's Signature**: ________________ **Date** : __ __ / __ __ __ / __ __ __ __<br>(DD / MMM / YYYY)	
**Reason(s) for screen failure:**	
1.	
2.	
3.	

Version 2

Date Modified: 22.08.2022
